# CXCR1 Depletion in Ly6C^+^ cDC2 Alleviates Acute Lung Injury via Modulation of Th17/Treg Balance

**DOI:** 10.1002/advs.202506287

**Published:** 2025-08-11

**Authors:** Shenghui Li, Wei Zhuang, Yang Wang, Hao Qin, Xichun Qin, Xianliang Yan, Xuefei Hu, Xiucheng Liu, Chang Chen

**Affiliations:** ^1^ Department of Thoracic Surgery Shanghai Pulmonary Hospital Tongji University School of Medicine Shanghai 200433 China; ^2^ Laboratory of Emergency Medicine Second Clinical Medical College of Xuzhou Medical University Xuzhou Jiangsu Province 221002 China; ^3^ Emergency Medicine Department of the Affiliated Hospital of Xuzhou Medical University Xuzhou Jiangsu Province 221002 China; ^4^ Department of Thoracic Surgery Shanghai Key Laboratory of Clinical Geriatric Medicine HuaDong Hospital Affiliated to Fudan University Shanghai 200041 China; ^5^ Shanghai Engineering Research Center of Lung Transplantation Shanghai 200433 China

**Keywords:** acute lung injury, CXCR1, Ly6C^+^ cDC2, T cell differentiation

## Abstract

Dendritic cells (DCs) play a critical role in the development of acute lung injury (ALI) / acute respiratory distress syndrome (ARDS), but the underlying mechanisms remain poorly understood, due to their heterogeneous phenotype and function. In this study, a novel DC subset is defined in mice, Ly6C⁺ cDC2, which corresponds to CD14⁺ cDC2 in humans. These subsets highly express C‐X‐C motif chemokine receptor 1 (Cxcr1) and exhibit pro‐inflammatory effects during ALI. Ex vivo, Ly6C⁺ cDC2s release higher levels of Il‐6 and Il‐1β, thereby promoting naïve T cells to differentiate into Th17 cells. Notably, Cxcr1 deficiency reduced the release of Il‐6 and Il‐1β from Ly6C⁺ cDC2s and shifted naïve T cells toward Treg differentiation, resulting in a decreased Th17/Treg ratio. In vivo, adoptive transfer of Ly6C⁺ cDC2s increased the Th17/Treg ratio in the lungs and spleens of LPS‐treated mice, exacerbating lung injury. Specific depletion of Cxcr1 in DCs significantly reduced the severity of ALI and mortality. Mechanistically, it is found that Cxcr1 regulates the expression of Il‐6 and Il‐1β in Ly6C⁺ cDC2s through the MEK1/ERK/NF‐κB pathway. Collectively, pro‐inflammatory Ly6C⁺ cDC2s are identified as key effector cells mediating the role of Cxcr1 signaling in modulating T cell differentiation, driving the progression of ALI.

## Graphical abstract

Pro‐inflammatory Ly6C⁺ cDC2s are identified as key effector cells mediating Cxcr1 signaling, driving the progression of ALI. Ly6C⁺ cDC2s release high levels of Il‐6 and Il‐1β, inducing Th17 differentiation. Cxcr1 deficiency reduces Il‐6 and Il‐1β production from Ly6C⁺ cDC2s through suppressing MEK1/ERK /NF‐κB signaling, thereby shifts naïve T cells towards Treg differentiation.

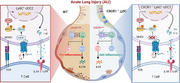

## 1 Introduction

Acute lung injury (ALI)/acute respiratory distress syndrome (ARDS) refers to a intricate clinical syndrome caused by a variety of pulmonary (e.g., aspiration, pneumonia) or non‐pulmonary (e.g., trauma, pancreatitis, sepsis) conditions, with a hospital mortality rate of 35–45% due to the lack of effective approved therapies.^[^ ^1^, ^2^, ^3^ ^]^ Regarding the pathogenesis, aberrant infiltration of immune cells combined with excessive release of pro‐inflammatory cytokines are the major factors driving the progression of ALI.^[^ ^4^ ^]^ This has led to an intense focus on the molecular mechanisms underlying the immunopathological changes in ALI in order to develop innovative and promising therapeutic strategies.

Previous findings have highlighted the aberrant activation of innate immune responses in ALI, particularly involving neutrophils and macrophages.^[^ ^5^, ^6^ ^]^ In contrast, recent studies have increasingly focused on elucidating the indispensable role of adaptive immune responses during both the initiation and resolution phases of ALI.^[^ ^7^, ^8^, ^9^ ^]^ In patients with ARDS related to trauma, bacterial infections, or coronavirus disease 2019 (COVID‐19), the activation of adaptive immune responses has been observed, especially an imbalance in the T helper 17 cell (Th17)/regulatory T cell (Treg) ratio.^[^ ^10^ ^]^ Kapur et al.^[^ ^11^ ^]^ also found that in vivo depletion of Tregs or dendritic cells (DCs) exacerbated antibody‐mediated ALI, which was mediated by reduced levels of interleukin‐10 (IL‐10). Therefore, the dysregulated Th17/Treg response is a critical hallmark of ALI and plays a pivotal role in determining the onsets and outcomes of ALI.

Dendritic cells (DCs) are considered central to coordinating adaptive immune responses due to their unique ability to initiate T cell responses and guide their differentiation into effector cells.^[^ ^12^, ^13^, ^14^, ^15^, ^16^ ^]^ In inflammatory conditions such as ARDS, COVID‐19, or transplant rejection, DCs continuously migrate to the lungs, where they help orchestrate local immune responses and regulate inflammatory outcomes. In murine models of ALI, the rapid accumulation of mature DCs in the lungs was also observed, and depletion of DCs led to increased mortality in mice.^[^ ^11^, ^17^, ^18^ ^]^ Notably, DCs are heterogeneous, consisting of multiple subpopulations that differ in phenotype, anatomical location, and function. The current classification of DC subsets relies on their cellular and molecular ontogeny: i) type 1 classical DCs (cDC1); ii) type 2 classical DCs (cDC2); iii) monocyte‐derived DCs (mo‐DCs), and iv) plasmacytoid DCs (pDCs).^[^ ^13^, ^19^, ^20^ ^]^ The cDC1 subset excels at presenting cell‐associated antigens to CD8^+^ T cells, thereby promoting a type I immune response.^[^ ^19^ ^]^ In contrast, the cDC2 subset primarily activates CD4^+^ T cell responses through MHC class II presentation.^[^ ^19^ ^]^ Moreover, cDC2 represent a heterogeneous population characterized by variable surface expression of Esam or CLEC12A, with distinct functional roles, indicating further diversification within the cDC2 subset.^[^ ^21^, ^22^ ^]^ Thus, it is vital to further explore the complexity of DC subsets and to unravel the detailed mechanisms through which they orchestrate adaptive immunity and regulate the onset and progression of ALI.

Our recent studies focused on the role of DCs in autoimmune diseases. We found that C‐X‐C motif chemokine receptor 1 (Cxcr1) signaling was abnormally activated in DCs within inflammation‐inducing experimental autoimmune encephalomyelitis (EAE) and ARDS. DC‐specific deletion of Cxcr1 significantly improved lung function in LPS‐treated mice and reduced the severity of EAE by reducing their Il‐6 and IL‐12p70 production.^[^ ^23^ ^]^ In fact, Cxcr1 and its ligands (such as Cxcl1, Cxcl2, and Cxcl8) have been highlighted in the pathogenesis of ALI, primarily through the recruitment of neutrophils and the regulation of endothelial redox homeostasis.^[^ ^24^ ^]^ In ARDS patients with COVID‐19 receiving mechanical ventilation, levels of Cxcl1, Cxcl5, and Cxcl8 are obviously upregulated.^[^ ^25^, ^26^ ^]^ Moreover, Cxcl8 concentrations in both circulation and bronchoalveolar lavage fluid (BALF) are significantly correlated with ARDS risk, disease severity, and clinical outcomes.^[^ ^24^, ^26^ ^]^ These findings suggest that Cxcr1 plays a key role in driving the onset and progression of ALI, highlighting its potential as a therapeutic target for the treatment of ALI.

This study identified elevated Cxcr1 expression in DCs within the lung tissues of both patients and animal models with ALI. Cxcr1 knockout and DC‐specific Cxcr1 knockout protect mice from lipopolysaccharide (LPS)‐induced lung injury, partially by regulating the Th17/Treg balance. Furthermore, we focused on a special subset of DCs (Ly6C^+^ cDC2) specifically responsible for driving Th cell differentiation, uncovering the detailed mechanisms by which they contribute to the progression and resolution of ALI.

## 2 Results

### 2.1 Cxcr1 is Highly Expressed in DCs and May Contribute to the Progression of ALI

In line with our previous findings,^[^ ^24^ ^]^ we observed that Cxcr1 deficiency mitigated LPS‐induced ALI, as evidenced by reduced lung injury scores, lower lung wet/dry weight ratio, as well as improved lung function and survival outcomes in mice (Figure S1A–G, Supporting Information). Moreover, BALF analysis revealed a reduction in leukocyte counts, total protein content, and levels of Il‐1β, Il‐6, and Il‐17a, accompanied by an elevation in Il‐10 levels in Cxcr1^−/−^ mice (Figure S1H–M, Supporting Information).

By reanalyzing single‐cell transcriptomic data from the GEO database (GSE137540), we found that CXCR1 was predominantly expressed in human neutrophils, monocytes, and DCs (Figure S2A, Supporting Information). We also observed a significant increase in the proportions of monocytes and DCs in the PBMCs of COVID‐19 patients with ARDS, along with upregulated CXCR1 gene expression (GSE150728; Figure S2B, Supporting Information). Similarly, increased Cxcr1 gene expression has also been detected in monocytes and DCs in experimental animal models of inflammation (GSE137540 and GSE165276; Figure S2C,D, Supporting Information). Flow cytometry and quantitative real‐time PCR (q‐PCR) further confirmed the upregulation of Cxcr1 expression in DCs within the lungs and spleens of LPS‐treated mice (Figure S2E–H, Supporting Information).

To exclude the involvement of Cxcr1 on macrophages and monocytes in the development of ALI, mice with a conditional myeloid‐specific Cxcr1 deletion (Cxcr1^fl/fl^LysM^cre^, referred to as Cxcr1^i△Mye/i△Mye^) and WT littermate controls (Cxcr1i^△Mye/+^) were constructed (Figure S3A, Supporting Information). We observed that mice with myeloid‐specific Cxcr1deficiency showed similar survival outcomes, lung function, lung injury scores, lung wet/dry weight ratio, as well as levels of leukocytes count and protein in BALF with WT mice (Figure S3B–I, Supporting Information). Additionally, Cxcr1^i△Mye/i△Mye^ mice showed similar Il‐6 and Il‐1β levels with WT mice, in regardless of LPS treatment. These findings suggest that elevated Cxcr1 expression in DCs may contribute to the initiation and progression of the ALI/ARDS instead of macrophages or monocytes.

### 2.2 Cxcr1 Knockout in DC Attenuates LPS‐Induced ALI

**Figure 1:**
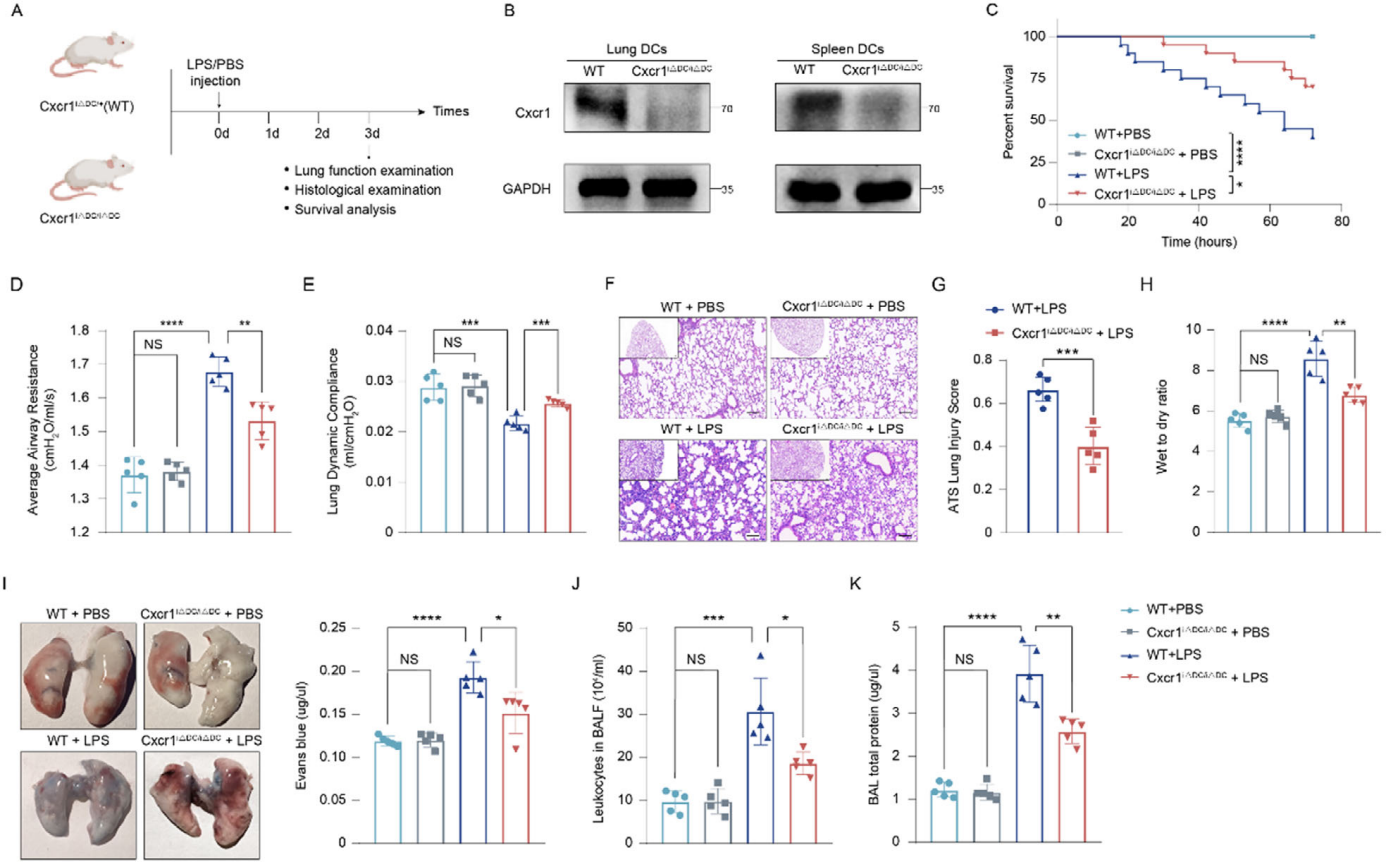
Cxcr1 depletion in DCs alleviates LPS‐induced ALI. A) Schematic diagram of inducible DC‐specific depletion of Cxcr1 and LPS‐induced ALI in mice. B) Verification of Cxcr1 efficiency in DCs isolated from the lungs and spleens of mice by western blot analysis. C) DC‐intrinsic Cxcr1 deficiency leads to long‐term survival in LPS‐treated mice (*n* = 20), log‐rank test. D,E) Determination of the lung average airway resistance (D) and dynamic compliance (E) (*n* = 5). F) Representative H&E staining of lung sections. Scale bars, 100 µm. G–K) DC‐intrinsic Cxcr1 deficiency reduces the lung injury score (G), lung wet/dry weight ratio (H), Evans blue leakage (I), and number of leukocytes (J) and total protein in BALF (K) (*n* = 5). *p < 0.05, **p < 0.01, ***p < 0.001,****p < 0.0001, NS, *p*>0.05 versus the indicated group, one‐way ANOVA followed by Tukey's post hoc test.

To investigate the in vivo role of Cxcr1 in DCs during ALI pathogenesis, mice with a conditional DC‐specific Cxcr1 deletion (Cxcr1^fl/fl^Itgax^cre^, referred to as Cxcr1^i△DC/i△DC^) and WT littermate controls (Cxcr1^i△DC/+^) were generated (**Figure** **1**A,B). Lung function and survival analyses exhibited improved outcomes in Cxcr1^i△DC/i△DC^ mice following LPS treatment compared to WT mice (Figure 1C–E). Histological examination showed reduced immune cells infiltration and alveolar septal thickening in Cxcr1^i△DC/i△DC^ mice, along with lower lung injury scores, wet/dry weight ratio, and Evans blue dye leakage (Figure 1F–I). Additionally, BALF analysis revealed a decrease in leukocyte counts and total protein levels in LPS‐treated Cxcr1^i△DC/i△DC^ mice (Figure 1J,K). These results suggest that DC‐intrinsic Cxcr1 deficiency significantly mitigates the severity of LPS‐induced ALI in mice.

### 2.3 DC‐Specific Cxcr1 Deficiency Decreased the Th17/Treg Ratio During ALI

**Figure 2:**
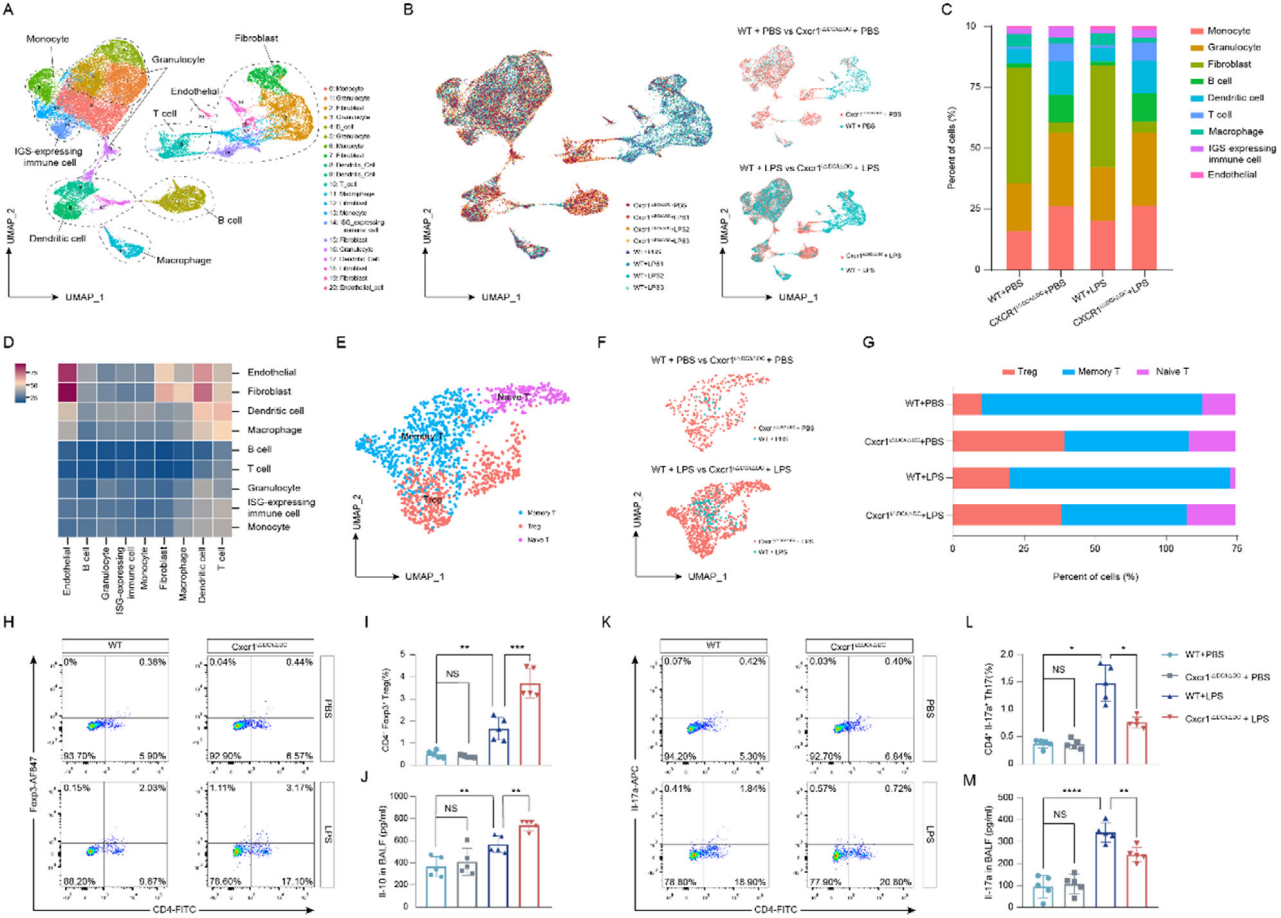
Cxcr1 deficiency in DCs reduces Th17/Treg ratio in the lungs of LPS‐treated mice. A) Uniform manifold approximation and projection (UMAP) plot of 32479 lung cells harvested from PBS‐treated WT mice, PBS‐treated Cxcr1^i△DC/i△DC^ mice, LPS‐treated WT mice, and LPS‐treated Cxcr1^i△DC/i△DC^ mice. B) UMAP visualization of cell populations colored according to sample source: WT (blue) or Cxcr1^i△DC/i△DC^ (red). C) The frequency of each cell type is depicted in the columns in PBS‐ or LPS‐treated WT and Cxcr1^i△DC/i△DC^ mice. D) Heatmap with double projection showing the cell‐cell interacting density among all identified cell subtypes, which is proportional to the number of ligands when isogenic receptors are expressed in the recipient cell type (blue, weak cell‐cell interactions; red, strong cell‐cell interactions). E,F) Clustering and annotating 1500 T cells (E), colored by sample source: WT (blue) or Cxcr1^i△DC/i△DC^ (red) (F). G) Fractions of T cell subpopulations in the lungs of PBS‐ or LPS‐treated WT and Cxcr1^i△DC/i△DC^ mice. H,I) Flow cytometry analysis showing the proportion of CD4⁺ Foxp3⁺ Tregs within the total leukocyte population in mouse lungs (*n* = 5). J) ELISA analysis detects Il‐10 levels in the BALF of mice with corresponding treatment (*n* = 5). K,L) Flow cytometry analysis showing the proportion of CD4^+^ Il‐17A^+^ Th17 cells within the total leukocyte population in mouse lungs (*n* = 5). M) ELISA analysis detects Il‐17A levels in the BALF of mice with corresponding treatment (*n* = 5). *p < 0.05, **p < 0.01, ***p < 0.001,****p < 0.0001, NS, p>0.05 versus the indicated group, one‐way ANOVA followed by Tukey's post hoc test.

To evaluate the consequences of DC‐specific Cxcr1 deficiency on inflammatory processes and immune equilibrium in lung tissues from LPS‐treated mice, single‐cell RNA sequencing (scRNA‐seq) analysis was performed (Figure S4A, Supporting Information). The quality‐controlled single‐cell atlas comprised 32479 cells, classified into 21 clusters, and annotated into 9 distinct cell types: monocytes, granulocytes, T cells, B cells, DCs, macrophages, interferon‐stimulated gene (ISG)‐expressing immune cells, fibroblasts, and endothelial cells (**Figure** **2**A; Figure S4B, Supporting Information). The 21 identified cell clusters were unevenly distributed across the 8 samples, with T and B cells being more enriched in the lung tissues of Cxcr1^i△DC/i△DC^ mice with PBS or LPS treatment (Figure 2B,C). Moreover, CellPhoneDB analysis revealed that, among all immune cells, the crosstalk between DCs and T cells was the most pronounced (Figure 2D). These findings suggest that DC‐specific Cxcr1 deficiency reshapes the adaptive immune landscape in mouse lung tissues during ALI.

Next, we focused on T cells and identified 5 distinct subtypes (Figure S4C, Supporting Information). Cluster 1 and 3 T cells were identified as Tregs based on high expression of Il2ra, Foxp3, and Fgl2. Cluster 0 and 2 were classified as memory T cells due to elevated expression of Ctsw, Ccl5, and Nkg7, while Cluster 4 were identified as naïve T cells for their specific expression of Lef1, Ccr7, and Sell (Figure 2E; Figure S4D, Supporting Information). Statistical analysis showed an increase in the proportion of Tregs in Cxcr1^i△DC/i△DC^ mice compared to the PBS‐treated and LPS‐treated WT mice (Figure 2F,G; Figure S4E, Supporting Information).

Flow cytometry analysis revealed that LPS treatment led to an increase in the proportion of CD4^+^Foxp3^+^ Tregs and CD4^+^Il‐17a^+^ Th17 cells in the lungs of mice. DC‐intrinsic Cxcr1 deficiency further increased the Treg proportion by almost 2.5‐fold and reduced the Th17 cell proportion to ≈50% of that seen in the lungs of LPS‐treated WT mice (Figure 2H,I,K,L), accompanied by elevated levels of IL‐10 and reduced concentrations of Il‐17a in the BALF (Figure 2J,M). Consistent results were also observed in the spleens of the mice (Figure S4F–I, Supporting Information). Overall, Cxcr1 deficiency in DCs decreased the Th17/Treg ratio in both the lungs and spleens of mice during ALI.

### 2.4 Cxcr1 Deficiency in DCs Inhibits Th17 Differentiation but Promotes Treg Differentiation

**Figure 3:**
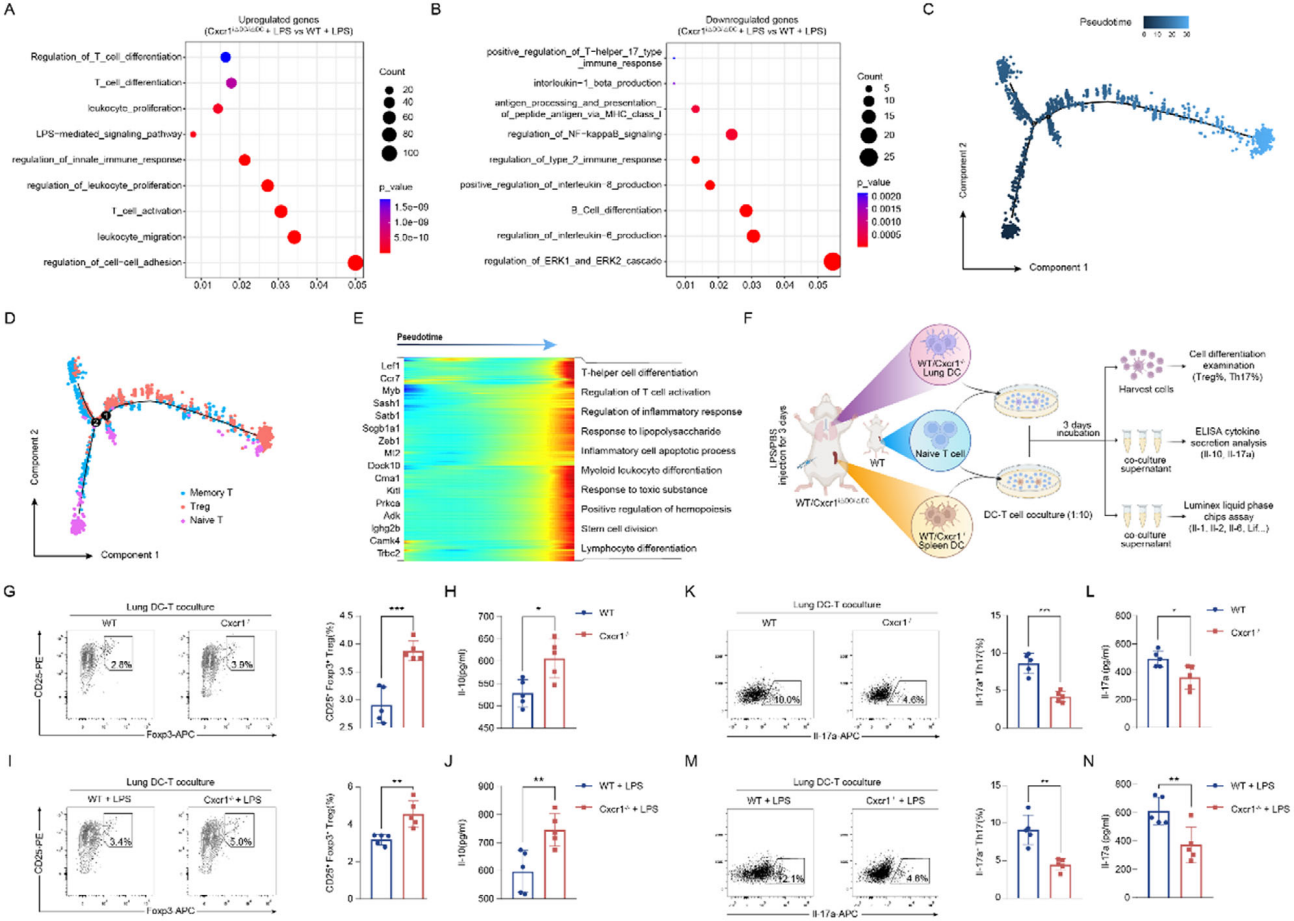
Cxcr1 deficiency in DCs inhibits Th17 cell differentiation but promotes Treg differentiation. A,B) Gene ontology (GO) enrichment analysis of significantly differentially expressed mRNAs in DCs between LPS‐treated Cxcr1^i△DC/i△DC^ mice and LPS‐treated WT mice, based on the scRNA‐seq data. C,D) The developmental trajectory of T cells inferred by Monocle2, colored by pseudotime (C) and cluster annotations (D). E) Heatmap revealed pseudotemporal expression pattern of genes among T cell populations. F) Schematic diagram of DC‐T cells co‐culture systems. G,I) Flow cytometry analysis showing the proportion of CD25^+^Foxp3^+^ Tregs from lung DC‐naïve T co‐culture system for Treg differentiation (*n* = 5). H,J) ELISA analysis detects IL‐10 levels in the co‐culture medium (*n* = 5). K,M) Flow cytometry analysis showing the proportion of IL‐17A^+^ Th17 cells from lung DC‐naïve T cells co‐culture system for Th17 differentiation (*n* = 5). L,N) ELISA analysis detects Il‐17 levels in the co‐culture medium (*n* = 5). *p < 0.05, **p < 0.01, ***p < 0.001, ****p < 0.0001, versus the indicated group, Student's T‐test.

Our scRNA‐seq data also indicated that the significantly differential expressed genes (DEGs, Cxcr1^i△DC/i△DC^ + LPS versus. WT + LPS) in DCs were enriched in pathways associated with “T_cell_differentiation”, “T_cell_activation”, and “positive_regulation_of_T‐helper_17_type_immune_response” (**Figure** **3**A,B). Next, we conducted trajectory analysis by mapping T cell clusters onto a pseudo‐time trajectory using the Monocle 2 algorithm (Figure 3C). The results indicated that more naïve T cells were induced to differentiate into Tregs at the terminal end of the trajectory in Cxcr1^i△DC/i△DC^ mice (Figure 3D; Figure S5A,B, Supporting Information). We further visualized DEGs co‐varying along the pseudotime trajectory to better understand the genetic characteristics of T cell clusters. The results indicated that “T cell differentiation” was linked to the upregulation of genes involved in “T cell activation” (Lef1, Ccr7, Myb) and “regulation of the inflammatory response” (Ighg2b, Scgb1a1, Cma1) (Figure 3E). These findings collectively suggest that Cxcr1 expression in DCs may influence the Th17/Treg balance by regulating T cell differentiation.

To validate this hypothesis, we established a DC‐naïve T cell co‐culture system in vitro (Figure 3F). Compared to the WT and WT + LPS groups, DCs from the lungs and spleens of Cxcr1^−/−^ mice preferentially promoted the naïve T cells to differentiate into CD25^+^Foxp3^+^ Tregs, which resulted in elevated IL‐10 levels in supernatant (Figure 3G–J; Figure S5C–F, Supporting Information). In contrast, Cxcr1 deficiency reduced the ability of DCs to promote Th17 cell differentiation, as evidenced by significantly decreased percentage of Il‐17a^+^ Th17 cells and production of Il‐17a in the co‐culture system (Figure 3K–N; Figure S5G–J, Supporting Information). Moreover, the addition of IL‐2 and Tgf‐β to the co‐culture medium to optimize Treg polarization conditions further amplified the capacity of Cxcr1‐deficient DCs to promote Treg differentiation (Figure S5K–R, Supporting Information). Taken together, these findings demonstrate that Cxcr1 deficiency in DCs promotes Treg differentiation while impairing Th17 differentiation in vitro.

### 2.5 Cxcr1 May Regulate the Trajectory of T Cell Differentiation by Reducing the Release of Il‐6 and Il‐1β in DCs

**Figure 4:**
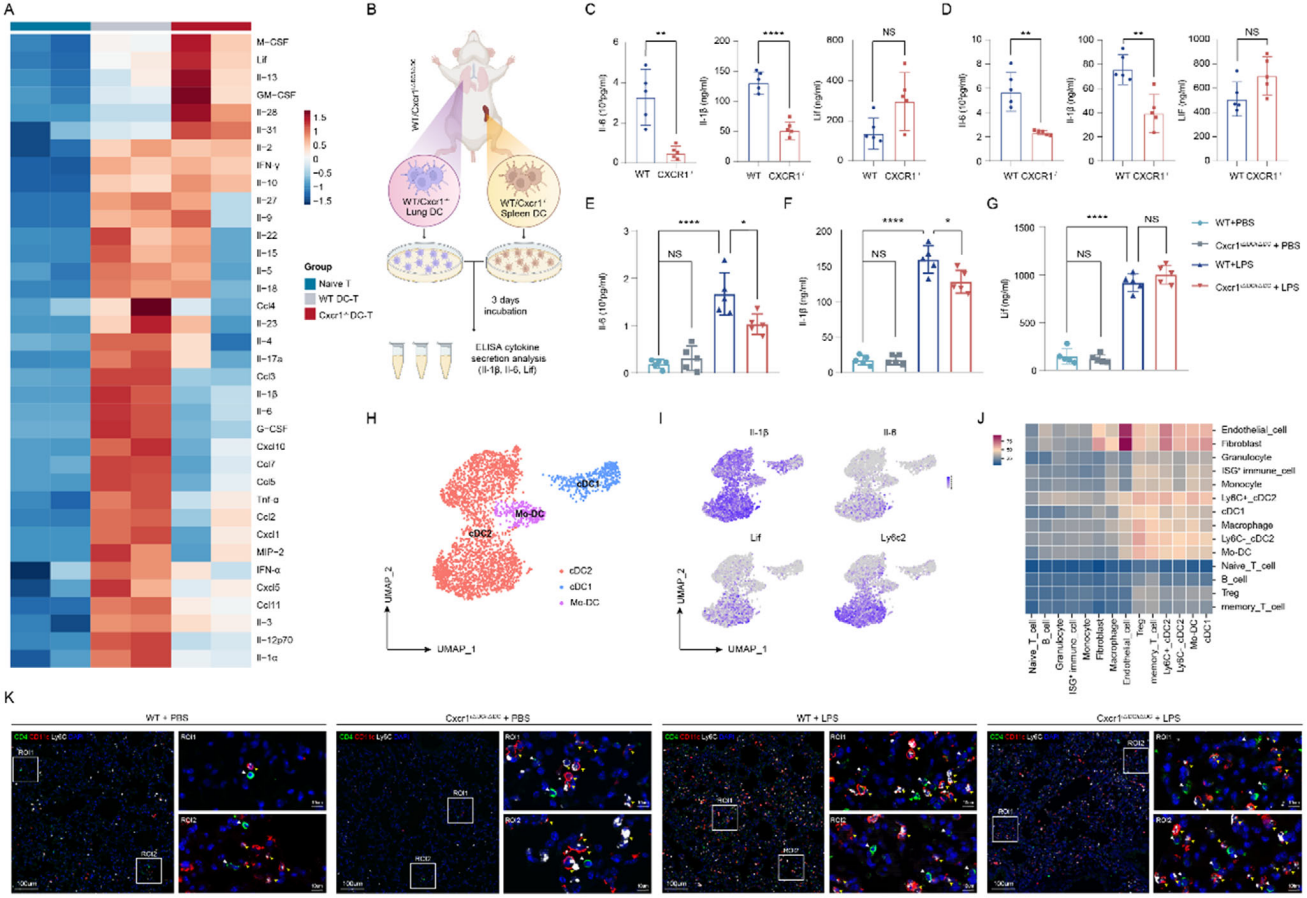
Identifying Ly6C^+^cDC2s as effector cells mediating the regulation of Cxcr1 on T cell differentiation. A) Luminex assay for detecting cytokines in the supernatants from naïve T cells cultured individually and co‐cultured with lung DCs (*n* = 2). B–D) ELISA analysis was used to evaluate the impact of Cxcr1 deficiency on the release of Il‐6, Il‐1β, and Lif of DCs from the lungs (C) and the spleens (D) (*n* = 5). **p < 0.01, ***p < 0.001, ****p < 0.0001, NS, p>0.05 versus the indicated group, Student's T‐test. (E‐G) Determinations of Il‐6 E), Il‐1β F), and Lif G) in BALF from PBS or LPS‐treated WT and Cxcr1^i△DC/i△DC^ mice (*n* = 5). *p < 0.05, **p < 0.01, ***p < 0.001, ****p < 0.0001, NS, p>0.05 versus the indicated group, one‐way ANOVA followed by Tukey's post hoc test. H) UMAP projections of subclustered DCs populations. Cells are colored by their annotations. I) Feature plots displaying the expression of Il‐6, Il‐1β, Lif, and Ly6c2 genes across DC clusters. J) Heatmap illustrating the density of cell‐cell interactions among DC subsets and other identified cell types. K) Representative immunofluorescence staining of Ly6C (white), CD11c (red), CD4 (green) and 4′,6‐diamidino‐2‐phenylindole (DAPI) (blue) in lung sections from PBS‐ or LPS‐treated WT and Cxcr1^i△DC/i△DC^ mice, Scale bar, 100 µm (left), 10 µm (right).

The above findings strongly suggest that Cxcr1 may regulate the differentiation of T cells by influencing DCs to produce and release specific exogenous cytokines. Therefore, a Luminex assay was conducted on the supernatants from naïve T cells cultured alone and the lung DC‐naïve T cell co‐culture system. In comparison to the supernatant from naïve T cells cultured alone, a total of 30 cytokines were upregulated in the supernatant of WT DC‐T cell co‐culture system, with 17 cytokines reversed by Cxcr1 deficiency. Additionally, 6 cytokines were significantly upregulated in the supernatant of the Cxcr1^−/−^DC‐naïve T cell co‐culture system (**Figure** **4**A). Among the altered 23 cytokines, leukemia inhibitory factor (Lif), Il‐1β, and Il‐6 were specifically highlighted for their established roles as key drivers of Th17/Treg cell differentiation.^[^ ^27^, ^28^, ^29^ ^]^

Subsequently, we assessed the activity of Il‐6, Il‐1β, and Lif released by DCs using ELISA analysis (Figure 4B). At the ex vivo level, Cxcr1 deficiency significantly reduced the release of Il‐1β and Il‐6 from DCs in the lungs and spleens (Figure 4C,D). Similarly, in vivo, DC‐specific Cxcr1 deficiency effectively reduced the LPS‐induced release of Il‐6 and Il‐1β in the BALF of mice (Figure 4E,F). Although the level of Lif was elevated in both the in vitro and in vivo situations, these differences were not statistically significant (Figure 4C,D,G). Based on these findings, we concluded that Cxcr1 deficiency in DCs mediated the differentiation trajectories of T cells mainly by reducing the Il‐6 and Il‐1β production.

### 2.6 Ly6C⁺ cDC2 Cells May Serve as Effector Cells Mediating the Regulation of T Cell Differentiation by Cxcr1

In fact, DCs are heterogeneous, consisting of multiple subpopulations that differ in phenotype and function.^[^ ^19^ ^]^ To identify the specific DC subpopulations involved in Cxcr1 regulation of cytokine secretion and T cell differentiation, we re‐clustered and annotated the DCs using scRNA‐seq data (Figure S6A, Supporting Information). Based on the differential expression of marker genes, the eight DC clusters were categorized into cDC1s, cDC2s, and mo‐DCs (Figure 4H; Figure S6B, Supporting Information). Further analysis revealed that Il‐6, Il‐1β, and Lif were primarily enriched in clusters 0 and 2 cDC2, which were characterized by high Ly6c, Il‐1α and Tgfbi expression (Figure 4I; Figure S6C, Supporting Information). We designated these cells as Ly6C⁺ cDC2s, likely corresponding to the “Ly6C⁺ cDC2‐like cells” previously identified by Gentaro et al., which accumulate in the lung tissues of mice exposed to inhaled indoor dust extract.^[^ ^30^ ^]^ CellPhoneDB analysis indicated that Ly6C⁺ cDC2s closely interact with T cells (Figure 4J). Immunofluorescence (IF) staining verified that CD11c^+^ Ly6C^+^ DCs were spatially located near CD4^+^ T cells in the lungs of mice (Figure 4K). The above findings led us to hypothesize that Ly6C⁺ cDC2 cells may serve as effector cells mediating the regulation of T cell differentiation by Cxcr1.

### 2.7 Cxcr1 Deficiency in Ly6C^+^ cDC2s Reduces the Production of Il‐6 and Il‐1β, and the Th17/Treg Ratio at the Ex Vivo Level

**Figure 5:**
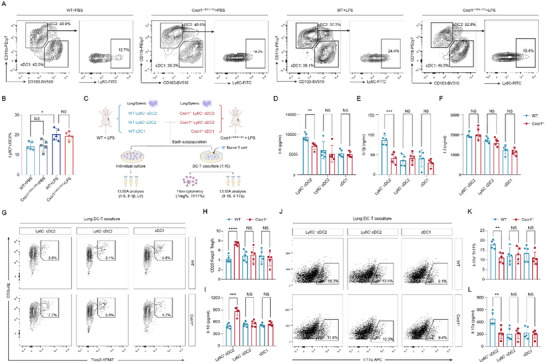
Cxcr1 deficiency in Ly6C^+^ cDC2s reduces the production of Il‐6 and Il‐1β and the Th17/Treg ratio at the ex vivo level. A,B) Flow cytometry analysis showing the proportion of Ly6C^+^ cDC2s in the lung tissues of mice with corresponding treatments (*n* = 5). *p < 0.05, **p < 0.01, ***p < 0.001, ****p < 0.0001, NS, p>0.05 versus the indicated group, one‐way ANOVA followed by Tukey's post hoc test. C) Schematic diagram of an individual culture and DC‐T cell co‐culture systems of DC subsets. D–F) ELISA analysis showing levels of Il‐6 (D), Il‐1β (E), and Lif (F) in supernatants from individually cultured Ly6C^+^ cDC2s, Ly6C^−^ cDC2s, and cDC1s isolated from the lungs of WT and Cxcr1^−/−^ mice treated with LPS (*n* = 5). G.H) Flow cytometry analysis in co‐culture models reveal the impact of Cxcr1 deficiency on the activity of Ly6C^+^ cDC2s, Ly6C^−^ cDC2s, and cDC1s in regulating the differentiation of naïve T cells into Tregs (*n* = 5). I) ELISA analysis detects IL‐10 levels in the co‐culture medium (*n* = 5). J,K) Flow cytometry analysis in co‐culture models reveal the impact of Cxcr1 deficiency on the activity of Ly6C^+^ cDC2s, Ly6C^−^ cDC2s, and cDC1s in inducing the differentiation of naïve T cells into Th17 cells (*n* = 5). L) ELISA analysis detects IL‐17A levels in the co‐culture medium (*n* = 5). **p < 0.01, ***p < 0.01, ***p < 0.001, ****p < 0.0001, NS, p>0.05 versus the indicated group, Student's T‐test.

Flow cytometry was subsequently applied to confirm the specific identification of Ly6C^+^ cDC2s in mouse lungs and spleens. In steady‐state lung tissue, Ly6C^+^ cDC2s comprised ≈10% of the total cDC2 population, which increased to ≈20% in LPS‐treated WT and Cxcr1^i△DC/i△DC^ mice (**Figure** **5**A,B). In contrast, the proportions of cDC1 and total cDC2 cells showed no significant differences in the lungs of mice receiving the corresponding treatments (Figure S6D,E, Supporting Information). In the spleen, the observed alterations in Ly6C⁺ cDC2 proportions paralleled those in the lungs; however, LPS treatment resulted in increased cDC1 proportions but decreased total cDC2 proportions, differing from the changes seen in the lungs (Figure S6F–I, Supporting Information).

We then isolated Ly6C⁺ cDC2s, Ly6C^−^ cDC2s, and cDC1 cells from the lungs and spleens of LPS‐treated mice using flow cytometry, followed by evaluation of their cytokine secretion and regulatory activity on naïve T cell differentiation at the ex vivo level (Figure 5C). Our results revealed that, compared to Ly6C^−^ cDC2s and cDC1 cells, Ly6C⁺ cDC2s release higher levels of Il‐6 and Il‐1β (Figure 5D,E; Figure S7A,B, Supporting Information), thereby increasing the Th17/Treg ratio by directing the differentiation of naïve T cells into Th17 cells in both the lungs (Figure 5G–L) and spleens (Figure S7D–I, Supporting Information). Notably, Cxcr1 deficiency diminished the release of Il‐6 and Il‐1β, but not Lif from Ly6C⁺ cDC2s (Figure 5D–F; Figure S7A–C, Supporting Information), which skewed naïve T cells towards Treg differentiation, resulting in a decreased Th17/Treg ratio (Figure 5G–L, Figure S7D–I, Supporting Information). Similarly, under optimized Treg polarization conditions, the ability of Cxcr1‐deficient Ly6C⁺ cDC2s to promote Treg differentiation was further amplified (Figure S7J–O, Supporting Information). By comparison, Ly6C^−^ cDC2s and cDC1s unbiasedly stimulate naïve T cells towards Treg or Th17 lineages, independent of Cxcr1 expression.

### 2.8 Cxcr1 Deficiency in Ly6C^+^ cDC2s Reduces the Production of Il‐6 and Il‐1β, and the Th17/Treg Ratio In Vivo

**Figure 6:**
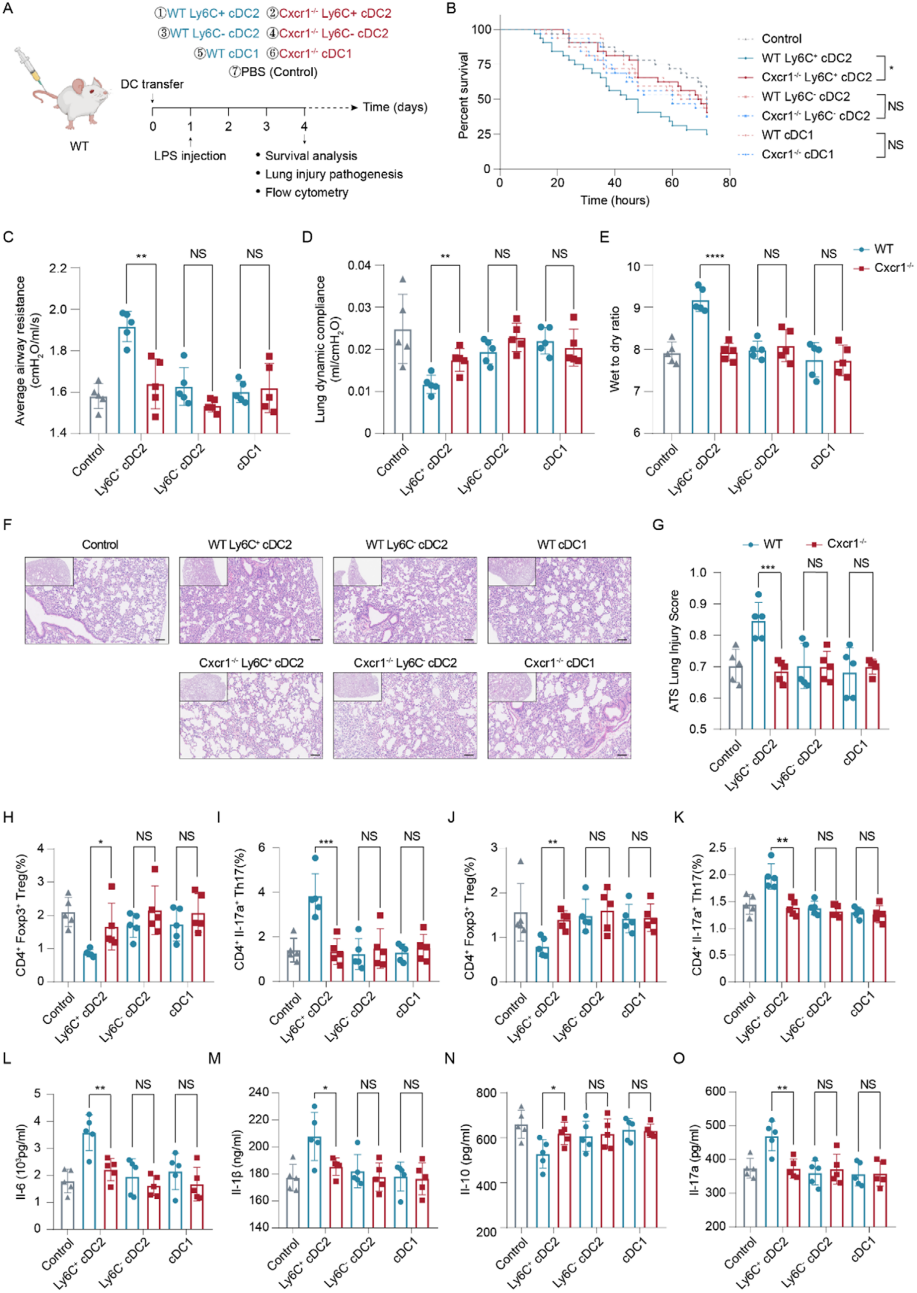
Cxcr1 knockout attenuates the proinflammatory effect of Ly6C^+^ cDC2 during LPS‐induced ALI. A) Experimental scheme of DCs transfer in LPS‐treated mice. B–G) Survival analysis (*n* = 20), log‐rank test (B), average airway resistance (C), lung dynamic compliance (D), wet‐to‐dry ratio (E), H&E staining of lung sections (F), and lung injury score (G) in LPS‐treated mice with PBS or DCs transfer. Scale bars, 100 µm. H–K) Percentage of lung Tregs (H), lung Th17 cells (I), spleen Tregs (J), and spleen Th17 cells (K) in LPS‐treated mice with PBS or DCs transfer (*n* = 5). L–O) ELISA analysis detects the levels of Il‐6 (L), Il‐1β (M), Il‐10 (N), and Il‐17A (O) in BALF from mice with corresponding treatment (*n* = 5). *p < 0.05, **p < 0.01, ***p < 0.01, ****p < 0.0001, NS, p>0.05 versus the indicated group, Student's T‐test.

Next, to verify the role of WT and Cxcr1‐deficient Ly6C⁺ cDC2 cells in regulating T cell differentiation in the context of ALI, we adoptively transferred Ly6C⁺ cDC2, Ly6C^−^ cDC2, and cDC1 cells derived from the spleens of WT and Cxcr1^−/−^ mice into recipient mice one day prior to LPS treatment (**Figure** **6**A). Mice receiving WT Ly6C⁺ cDC2 cells exhibited significantly worsened survival outcomes and pulmonary function, along with increased wet‐to‐dry weight ratio and lung injury scores, compared to the control mice or mice receiving equally viable Cxcr1‐deficient Ly6C⁺ cDC2 (Figure 6B–G). Unsurprisingly, neither Cxcr1‐deficient nor WT cDC1s or Ly6C^−^ cDC2s had any significant impact on lung injury or pulmonary function in the recipient mice (Figure 6B–G). Regarding the effect on T cell differentiation, mice receiving WT Ly6C⁺ cDC2s exhibited a decreased Tregs percentage but an increased Th17 cells proportion in both the lungs and spleens compared to the control mice or those receiving equally viable Cxcr1‐deficient Ly6C⁺ cDC2s (Figure 6H–K), accompanied by elevated IL‐10 while decreased Il‐17a, Il‐6, and Il‐1β concentration in BALF (Figure 6L–O). Ly6C^−^ cDC2s and cDC1s, whether Cxcr1‐deficient or not, transferred into the recipient mice had minimal effects on the Th17/Treg ratio in the lung and spleen tissues, as well as on the levels of Il‐6, Il‐1β, IL‐10, and IL‐17A in BALF (Figure 6H–O). Integrating our in vivo and in vitro findings, it is evident that, compared to other cDC subpopulations, Ly6C⁺ cDC2s exhibit elevated expression of Il‐6 and Il‐1β, driving naïve T cells towards pro‐inflammatory Th17 differentiation. Cxcr1 specifically regulates the release of Il‐6 and Il‐1β from the Ly6C⁺ cDC2 subpopulation, thereby influencing the T cell differentiation and Th17/Treg balance.

### 2.9 The MEK1/ERK/NF‐κB Signaling Pathway Mediates the Role of Cxcr1 in Regulating T Cell Differentiation in Ly6C⁺ cDC2

**Figure 7:**
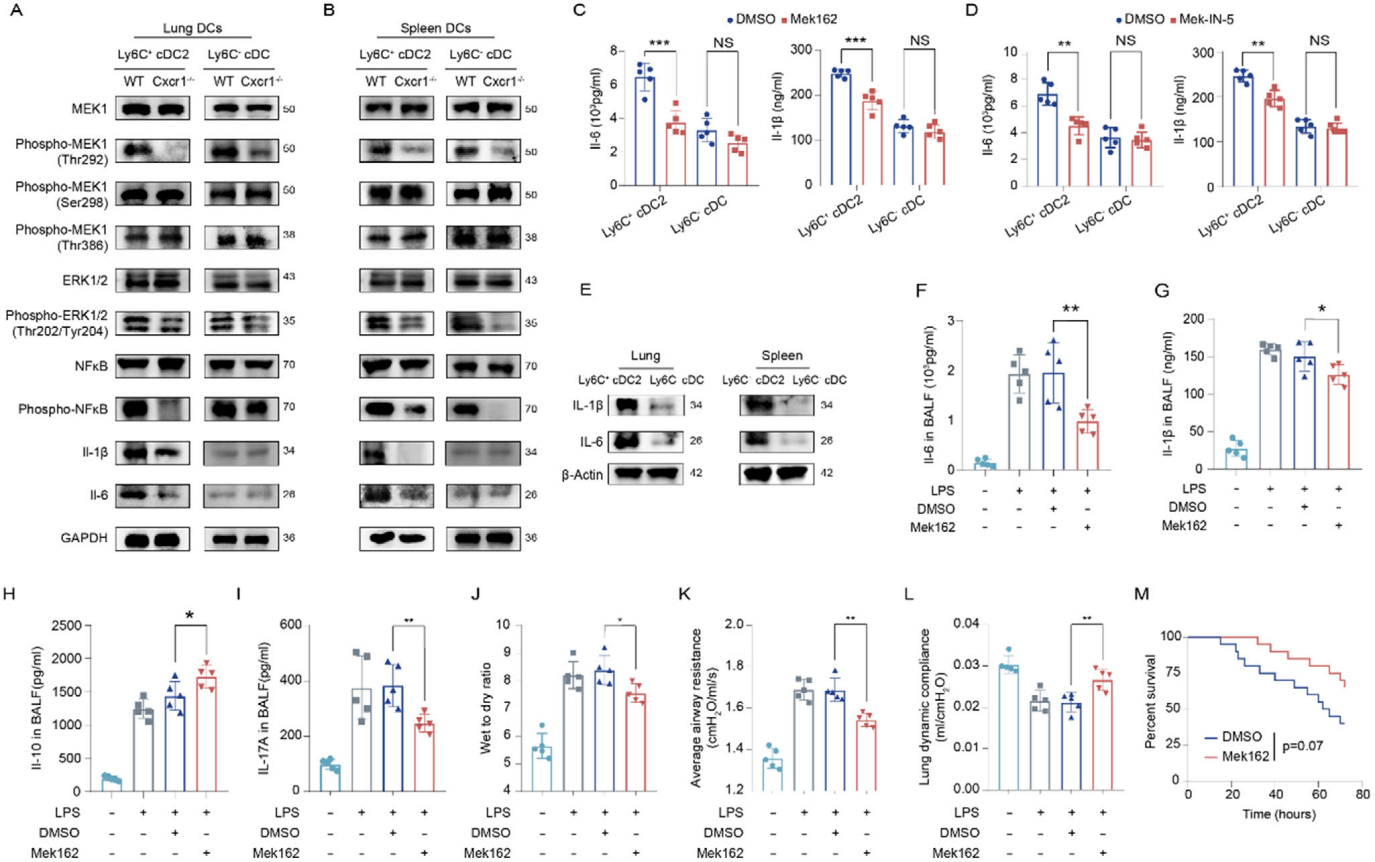
The MEK1/ERK/NF‐κB signaling pathway mediates the role of Cxcr1 in regulating T cell differentiation in Ly6C⁺ cDC2. A,B) Western blot analysis showing that Cxcr1 deficiency leads to downregulation of the MEK1/ERK/NF‐kB axis in Ly6C⁺ cDC2s isolated from the lungs and spleens of LPS‐treated mice. C,D) ELISA analysis detects the effect of the MEK1 inhibitor MEK162 (C) and pMEK/pERK inhibitor MEK‐IN‐5 (D) on the secretion of Il‐6 and Il‐1β by Ly6C⁺ cDC2s and Ly6C^−^ cDC2s isolated from the lungs of LPS‐treated mice. (*n* = 5). *p < 0.05, **p < 0.01, ***p < 0.001, ****p < 0.0001, NS, p>0.05 versus the indicated group, Student's T‐test. E) Baseline expression of Il‐6 and Il‐1β by Ly6C⁺ cDC2s and Ly6C‐ cDC2s. F–I) ELISA analysis detects the effect of MEK162 on the levels of Il‐6 (F), Il‐1β (G), Il‐10 (H), and Il‐17A (I) in BALF (*n* = 5). J–L) Effects of Mek162 on the lung wet/dry weight ratio (J), average airway resistance (K), and lung dynamic compliance (L) (*n* = 5). *p < 0.05, **p < 0.01, ***p < 0.001, ****p < 0.0001, NS, p>0.05 versus the indicated group, one‐way ANOVA followed by Tukey's post hoc test. M) Effect of Mek162 on the survival of mice with LPS treatment (*n* = 20), log‐rank test.

Lastly, we sought to elucidate the potential mechanisms through which Cxcr1 regulates the production of Il‐6 and Il‐1β in Ly6C⁺ cDC2s, thereby impacting T cell differentiation. Our previous studies have shown that high levels of Cxcr1 serve as potent activators of the MEK/ERK signaling pathway in endothelial cells.^[^ ^24^ ^]^ Furthermore, GO analysis in DCs revealed downregulation of ERK and NF‐κB signaling in LPS‐treated Cxcr1^i△DC/i△DC^ mice (Figure 3B). Therefore, we investigate whether this regulatory mechanism also applies to Ly6C⁺ cDC2s. Using Ly6C^−^ cDCs as controls, western blot analysis indicated that Cxcr1 deficiency resulted in reduced phosphorylation of MEK1 at Ser292 (phospho‐Ser292‐MEK1), and a decrease in ERK1/2 phosphorylation, along with diminished phosphorylation of NF‐kB, a downstream target of the MEK/ERK pathway, in both Ly6C⁺ cDC2s and Ly6C^−^ cDCs from the lungs and spleens of mice (**Figure** **7**A,B). Inhibiting phosphorylation of MEK1 and ERK significantly decreased the Il‐6 and Il‐1β production from Ly6C⁺ cDC2s (Figure 7C,D). Notably, Il‐1β and Il‐6 are expressed at very low levels in Ly6C^−^ cDCs, rendering them largely unaffected by Cxcr1 deficiency or MEK/ERK signaling inhibition (Figure 7A–D). WB results comparing the Il‐6 and IL‐1β expression levels between Ly6C⁺ cDC2s and Ly6C^−^ cDCs further validates the lower baseline expression of these two cytokines in the Ly6C^−^ cDCs (Figure 7E). These observations help to explain why Cxcr1 expression is particularly essential for the functional activity of Ly6C⁺ cDC2s instead of Ly6C^−^ cDCs. In vivo studies demonstrated that suppression of MEK1/ERK1 signaling with Mek162 led to a significant decrease in levels of pro‐inflammatory cytokines, including Il‐6, Il‐1β, and Il‐17a, while increasing the anti‐inflammatory cytokine Il‐10 in the BALF (Figure 7F–I). Furthermore, treatment with the MEK1/ERK1 inhibitor Mek162 significantly alleviated LPS‐induced lung injury and improved lung function, although the observed improvement in survival outcomes did not reach statistical significance (Figure 7J–M). Collectively, Cxcr1 knockdown specifically suppressed the Il‐6 and Il‐1β production in Ly6C^+^ cDC2 via MEK/ERK/NF‐kB pathway downregulation.

### 2.10 CXCL5 Serves as the Primary Ligand Activating CXCR1 Signaling in Mouse DCs

To identify the main ligands of Cxcr1 in mouse DCs, we evaluate the effects of conventional ligands including Cxcl1, Cxcl2, Cxcl5, and Cxcl6 on the production of Il‐6 and Il‐1β by DCs in ex vivo setting. Notably, Cxcl8 was not included in investigation due to the absence of a direct homologous gene in mice. Lung DCs were sorted and cultured with these exogeneous ligands, and then Il‐6 and Il‐1β secretion was examined. After 48 hours, Cxcl1, Cxcl2, and Cxcl6 showed no significant effect on Il‐6 or IL‐1β levels. In contrast, Cxcl5 significantly increased Il‐6 and Il‐1β production from DCs, while this effect was abolished by Cxcr1 depletion (Figure S8A,B, Supporting Information). These findings identify Cxcl5 as the primary ligand activating Cxcr1 signaling in mouse DCs.

To investigate potential crosstalk between DCs and T cells, we sorted lung T cells and cultured them with exogenous Il‐6 and IL‐1β. After 48 h, ELISA analysis revealed no significant change in Cxcl5 production by T cells in response to these two cytokines (Figure 3C). These results suggest the absence of a feedback loop between DCs and T cells mediated by the Cxcl5/Cxcr1 axis.

### 2.11 Identification of a Novel cDC2 Subset in Human

Comparative transcriptomics and functional studies have shown a correspondence between mouse and human cDC subsets:^[^ ^19^, ^20^, ^31^ ^]^ i. human lung tissues contain CD1c^hi^ cross‐presenting DCs with functional homology to mouse CD11b^+^ nonlymphoid DCs;ii. novel insights into the relationships between dendritic cell subsets in human and mouse revealed by genome‐wide expression profiling. Researches from the Bosteels et. al^[^ ^30^ ^]^ and Dutertre et. al^[^ ^32^ ^]^ suggests that CD14⁺ cDC2s in humans analogous to the Ly6C^+^ cDC2s subset identified in mice.

**Figure 8:**
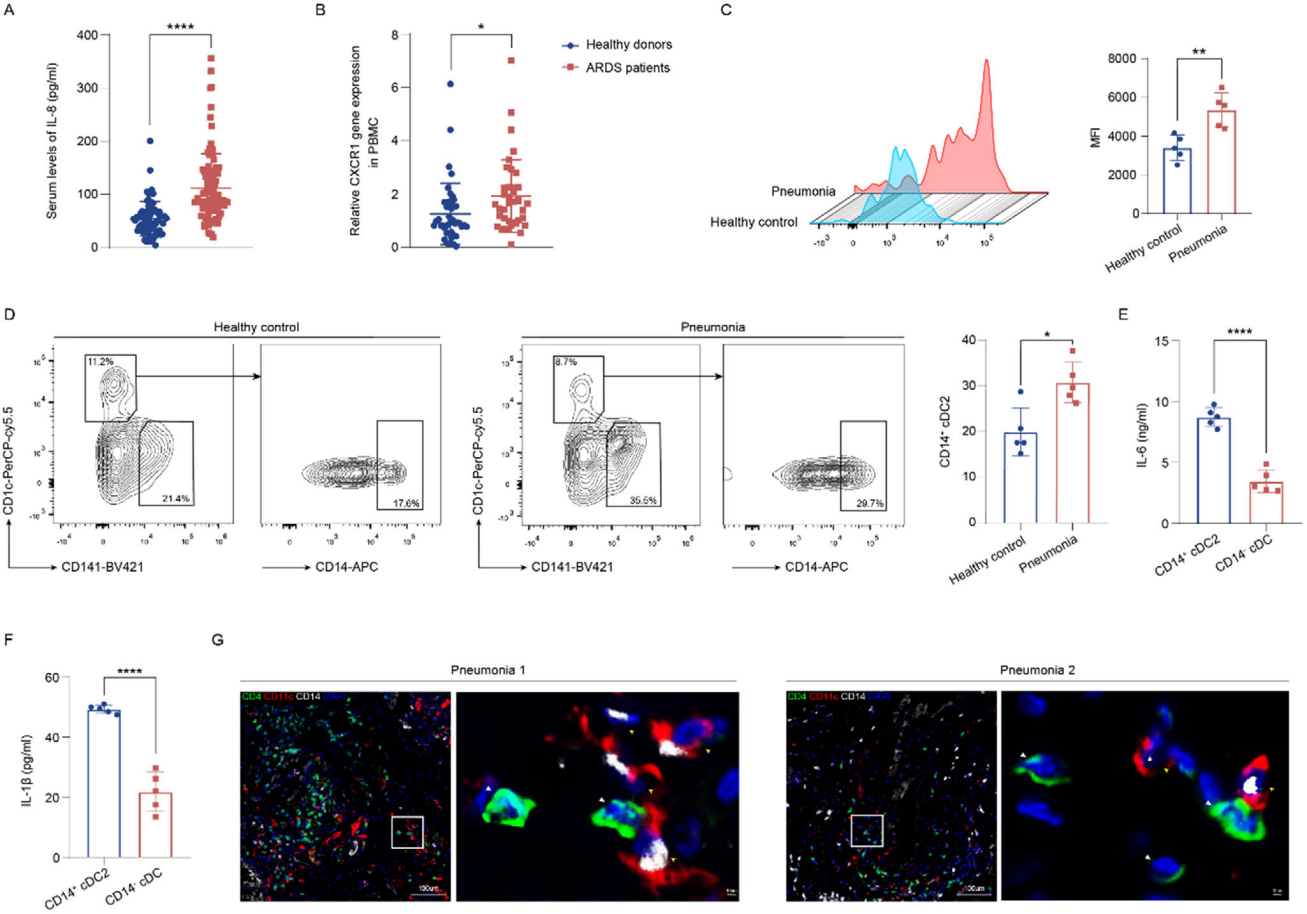
Identification of a novel cDC2 subset in human. A) ELISA analysis detects IL‐8 levels in the serum of ARDS patients and healthy donors. B) PCR determined CXCR1 gene expression in PBMC of ARDS patients and healthy donors. C) Flow cytometry analysis of CXCR1 expression in lung DCs of pneumonia patients and healthy controls (*n* = 5). D) Flow cytometry analysis of CD14^+^ cDC2 proportion in lungs of pneumonia patients and healthy controls (*n* = 5). E,F) ELISA analysis of IL‐6 (E) and IL‐1β (F) production of lung CD14^+^ cDC2 and CD14^−^ cDC (*n* = 5). G) Representative immunofluorescence staining of CD14 (white), CD11c (red), CD4 (green), and DAPI (blue) in lung sections from pneumonia patients, Scale bar, 100 µm (left), 10 µm (right) *p < 0.05, **p < 0.01, ***p < 0.01, ****p < 0.0001, NS, p>0.05 versus the indicated group, Student's T‐test.

Next, we sought to investigate the role of CXCR1 signaling and CD14⁺ cDC2s in humans. Peripheral blood was collected from 97 ARDS patients and 72 healthy donors, with baseline information displayed in Table S2 (Supporting Information). Our data confirmed the activation of IL‐8/CXCR1 signaling in the peripheral blood of ARDS patients (**Figure** **8**A,B). Given that ARDS is not typically an indication for lung transplantation or biopsy, obtaining lung tissues from ARDS patients to assess the expression of CXCR1 on pulmonary DCs is challenging. As an alternative, we obtained lung tissues from three patients with pulmonary hypertension complicated by pneumonia and two patients with interstitial lung disease complicated by pneumonia after lung transplantation. Additionally, lung tissues distant from the tumor site were collected from five early‐stage lung cancer patients who had undergone surgical resection as healthy controls. Flow cytometry analysis showed that CXCR1 expression was significantly upregulated in the lung DCs of pneumonia patients (Figure 8C). Moreover, the proportion of CD14⁺ cDC2s in the lungs of pneumonia patients increased by nearly 1.5‐fold compared with healthy controls (Figure 8D). Ex vivo analysis revealed higher levels of IL‐6 and IL‐1β production in CD14⁺ cDC2s compared to CD14^−^ cDCs (Figure 8E,F). Immunofluorescence staining analysis showed that CD11c⁺ CD14⁺ DCs were spatially located near CD4⁺ T cells in the lungs of pneumonia patients (Figure 8G). These results highlight the essential role of IL‐8/CXCR1 signaling in ARDS and demonstrate that human CD14⁺ cDC2s display similar phenotypic characteristics and localization patterns to Ly6C⁺ cDC2s.

## 3 Discussion

In this study, we defined a novel DC subset, present in both mice (Ly6C⁺ cDC2) and humans (CD14^+^ cDC2), which plays a crucial role in mediating Th17/Treg balance under inflammatory conditions. Our in vivo and in vitro findings reveal that Cxcr1 deficiency specifically inhibits Il‐6 and Il‐1β production in Ly6C⁺ cDC2 cells. This suppression reduces Th17 differentiation while promoting Treg differentiation of naïve T cells, thereby alleviating LPS‐induced ALI, improving pulmonary function, and enhancing survival outcomes in mice.

Classical studies have emphasized the swift mobilization and accumulation of DCs in response to lung injury, suggesting their crucial roles in coordinating immune responses to pulmonary challenges.^[^ ^14^, ^17^, ^18^, ^33^, ^34^ ^]^ DCs serve as the first line of immunological defense and have diversified into various subsets distributed across the body. In both humans and mice, lung cDCs are classically categorized into cDC1s and cDC2s, each exhibiting a distinct division of labor.^[^ ^19^ ^]^ Unlike cDC1s, the cDC2 subset is a more heterogeneous population that remains poorly defined. Several studies have employed new single‐cell technologies to further annotate cDC2 subpopulations and characterize their identities and functions. Brown et al.^[^ ^22^ ^]^ proposed categorizing the cDC2 subset into cDC2a (Tbx21^+^) and cDC2b (Rorc^+^), with the latter displaying strong pro‐inflammatory potential. Lewis et al.^[^ ^21^ ^]^ identified a distinct population within CD11b⁺ cDC2s in the spleens of mice, characterized by high expression of adhesion molecules Esam (Esam^hi^ cDC2), which proliferates in situ and promotes the activation of CD4⁺ T cells. In this study, we identified a distinct cluster of cDC2s in both mice and humans, with a functional capacity to produce high levels of pro‐inflammatory cytokines such as Il‐6 and Il‐1β, characterized by specific Ly6C and CD14 expression, respectively. This cluster may correspond to previously reported “Ly6C⁺ cDC2‐like cells” that accumulate in the lung tissues of mouse models of asthma,^[^ ^30^ ^]^ or “CD14⁺ CD163⁺ DC3s” that expand in the peripheral blood of patients with systemic lupus erythematosus.^[^ ^32^ ^]^ Thus, Ly6C⁺ cDC2s in mice, which map to CD14^+^ cDC2s in humans, represent a stable cell lineage. and their biological activity, as well as their roles in the development and diseases warrant further investigation.

In adaptive immunity, cDC1s are highly efficient at cross‐presenting antigens to CD8⁺ T cells, whereas cDC2s undertake a broader range of functions, such as presenting antigens to CD4⁺ T cells and promoting T cell memory.^[^ ^19^ ^]^ Treg and Th17 cells are key CD4⁺ T cell subsets involved in inflammatory responses, homeostasis regulation, and pathogen clearance. Th17 cells exert pro‐inflammatory effects primarily through Il‐17 secretion, while Treg cells counterbalance inflammation by releasing cytokines including IL‐10 and TGF‐β, thereby inhibiting Th17 responses.^[^ ^9^, ^35^, ^36^ ^]^ Emerging evidence indicates that Tregs are vital for suppressing pulmonary inflammation and promoting tissue repair and regeneration.^[^ ^11^, ^37^ ^]^ In patients, a higher Th17/Treg ratio in BALF is regarded as a negative prognostic factor for ARDS.^[^ ^10^ ^]^ In this study, we propose that Ly6C⁺ cDC2 is a core mediator in balancing Th17 and Treg cells within the organism. Using an in vitro co‐culture model, we found that Ly6C⁺ cDC2s induce naïve T cells to preferentially differentiate into the Th17 lineage by producing high levels of Il‐6 and Il‐1β. In vivo, the adoptive transfer of Ly6C⁺ cDC2s resulted in an increased Th17/Treg ratio in the lungs and spleens of mice following LPS treatment, leading to exacerbated lung injury and deterioration of lung function. These findings suggest that Ly6C⁺ cDC2s are distinct from Ly6C^−^ cDC2s and cDC1s in regulating T cell differentiation, highlighting the potential of Ly6C⁺ cDC2s as therapeutic targets for ALI.

Cxcr1 is widely recognized for its role in neutrophil chemotaxis and in regulating intracellular redox balance. It is strongly expressed in mammalian myeloid and endothelial cells, and is highly conserved across species.^[^ ^24^, ^38^, ^39^, ^40^, ^41^, ^42^ ^]^ Our previous research^[^ ^23^, ^24^ ^]^ demonstrated that genetic deletion of Cxcr1 in mice confers protection against LPS‐ or hyperoxia‐induced ALI. Blocking Cxcr1/2 signaling with Cxcr1 antagonists, ELR⁺ chemokine analogues, or antibodies has been explored as a therapeutic approach in preclinical ALI models.^[^ ^38^, ^39^, ^40^ ^]^ In humans, only a few studies have tested the effects of CXCR1 signaling inhibition on ARDS treatment.^[^ ^43^, ^44^ ^]^ In a Phase 2 clinical trial including 56 patients with COVID‐19‐related ARDS, reparixin, a non‐competitive allosteric inhibitor of CXCR1 and CXCR2, was compared to standard care. The incidence of adverse events—including worsening respiratory failure, need for ICU admission, mechanical ventilation, or death—was significantly lower in the reparixin group (16.7% [95% CI, 6.4‐32.8%] versus 42.1% [95% CI, 20.3‐66.5%], p = 0.02).^[^ ^43^ ^]^ The subsequent Phase 3 study enrolled 279 COVID‐19 patients hospitalized for respiratory failure showed a numerically higher survival without respiratory failure in the reparixin group, though not statistically significant, likely due to improved population immunity and COVID‐19 treatments.^[^ ^44^ ^]^ Here, we report the elevated expression of CXCR1 in the lung tissues of patients with pulmonary infections and LPS‐treated mice, particularly within DCs. Specific depletion of Cxcr1 in DCs significantly mitigated the severity of lung injury, improving gas exchange, capillary permeability, lung edema, neutrophil accumulation, and mortality. Our data also confirmed that Cxcr1 deficiency inhibited the secretion of Il‐6 and Il‐1β from Ly6C⁺ cDC2s, resulting in reduced Th17 differentiation. Additionally, we observed that Cxcr1 deficiency in Ly6C⁺ cDC2s facilitated the differentiation of naïve T cells into Tregs, potentially due to feedback inhibition of Th17 differentiation or a modest increase in Lif, a potent factor that promotes Treg differentiation.^[^ ^27^, ^28^ ^]^ Interestingly, these activities associated with Cxcr1 deficiency were not observed in other cDC subsets, indicating that Ly6C⁺ cDC2s, rather than other cDC populations, exert pro‐inflammatory effects and drive the progression of ALI in a Cxcr1‐dependent manner.

In view of the tissue niches‐specific differentiation of DCs, we investigated whether Ly6C^+^ cDC2s were present and regulated by Cxcr1 in spleen. Our results observed rapid aggregation of splenic Ly6C^+^ cDC2s under inflammatory conditions. Consistent effects of Cxcr1 deficiency on the production of cytokines and induction of Th‐cell differentiation were observed in vitro across lung and splenic cDC subpopulations. Adoptive transfer of splenic cDC subpopulations with different Cxcr1 status demonstrated that Cxcr1 knockout efficiently relieved the pro‐inflammatory impact of Ly6C^+^ cDC2s during ALI in vivo. These results demonstrate that Ly6C^+^ cDC2s are present across tissues with the same function and molecular regulatory mechanism in inflammatory settings. Considering that an ALI mouse model induced by intraperitoneal injection of LPS was also employed to induce sepsis, our results suggested that targeting Cxcr1 on splenic Ly6C^+^ cDC2s might be a valid strategy for the treatment of systemic inflammatory responses.

Although previous studies have linked the activation of the MEK1/ERK signaling axis to the inflammatory response in ALI,^[^ ^24^ ^]^ its specific role in controlling the plasticity of DCs subsets has yet to be fully elucidated. This study provides evidence that Cxcr1 deficiency downregulates the MEK/ERK/NF‐kB signaling axis in Ly6C^+^ cDC2s and decreases their Il‐6 and Il‐1β expression levels across different tissues in inflammatory conditions, underscoring the enormous contribution of this pathway to driving the pro‐inflammatory phenotype of Ly6C^+^ cDC2s. Notably, the expression of Il‐6 and Il‐1β were not altered in Cxcr1^−/−^ Ly6C^−^ cDCs, even though the MEK1/ERK/NF‐kB signaling axis was inactivated. This phenomenon might be attributed to the intrinsic lower expression of Il‐6 and Il‐1β in Ly6C^−^ cDCs, resulting in a less pronounced decline. Further studies need to be conducted to fully understand the discrepancy in the regulation of inflammatory cytokines between Ly6C^+^ cDC2s and Ly6C^−^ cDCs. The potent immunosuppressive and injury‐mitigating effects of the MEK inhibitor Mek162 indicate that targeting the CXCR1/MEK1/ERK signaling axis is an effective strategy to restore the Th17/Treg balance in ALI, which provides evidence for exploring novel treatments.

Although Cxcr1 was demonstrated to play an essential role in regulating the function of Ly6C^+^ cDC2s during LPS‐induced ALI, it is acknowledged that the LPS model may not fully recapitulate the complexity of viral ARDS, such as COVID‐19, which is characterized by pulmonary edema, hyaline membrane formation, and capillary congestion and microthrombosis.^[^ ^45^, ^46^ ^]^ Nonetheless, the LPS model remains highly relevant for investigating cytokine‐driven lung injury, a hallmark of sepsis/toxemia and ARDS triggered by severe systemic infections. This model is particularly well‐suited for studying the role of DCs – key regulators of inflammatory mediator secretion and adaptive immunity initiation – in the context of hyperinflammation. Our findings specifically illuminate CXCR1's function within this cytokine cascade. To directly address CXCR1's role in viral ARDS like COVID‐19, future research should employ clinically relevant models, such as microbial infection (e.g., viral pneumonia) and ventilator‐induced lung injury (VILI). Such studies will be crucial for translating our mechanistic insights into targeted therapies for viral ARDS.

In summary, our results underscore the significant role of pro‐inflammatory Ly6C⁺ cDC2s in secreting Il‐6 and Il‐1β as well as regulating the Th17/Treg balance in both humans and mice. Ly6C⁺ cDC2s were identified as key effector cells mediating the Cxcr1 signaling, which drives the progression of ALI. In contrast, Ly6C‐ cDCs exert minimal effects on both T cell differentiation and ALI development due to their low intrinsic expression of Il‐6 and Il‐1β, although the MEK/ERK/NF‐κB signaling is not inactivated during pulmonary inflammation. These findings deepen our understanding of the pathogenesis of ALI/ARDS, providing more precise therapeutic targets and intervention mechanisms for treating ALI.

## 4 Experimental Section

### Mice

C57BL/6J mice aged 6–8weeks were obtained from Gempharmatech Co. Ltd. Cxcr1‐deficient (Cxcr1^−/−^) mice were constructed through TALEN‐mediated gene targeting. The Cxcr1^flox/flox^ mice were crossed with LysM‐cre C57BL/6J mice to obtain myeloid‐specific Cxcr1 knockout mice (LysM‐creCxcr1^flox/flox^ mice), which were referred to as Cxcr1^i△Mye/i△Mye^ mice. The Cxcr1^flox/flox^ mice were crossed with Itgax‐cre C57BL/6J mice to obtain DC‐specific Cxcr1 knockout mice (Itgax‐creCxcr1^flox/flox^ mice), which were hereafter referred to as Cxcr1^i△DC/i△DC^ mice. All mice were housed in Tongji University (Shanghai, China) in specific pathogen‐free (SPF) context. The mice experiments were approved by the Ethics Committee of Shanghai Pulmonary Hospital (K25‐545).

### LPS‐Induced ALI Mouse Model

WT C57BL/6, Cxcr1^−/−^, and Cxcr1i^△DC/i△DC^ mice aged 6–8weeks were administered an intraperitoneal injection of a sublethal dose of LPS (10 mg kg^−1^; Sigma‐Aldrich, USA) or an equal volume of PBS as control.^[^ ^47^ ^]^ Mice were sacrificed 3 days post‐exposure for subsequent ScRNA sequencing, lung injury severity assessment, and inflammatory response analysis.

### Pulmonary Function Test

The Electro‐Medical Measurement Systems (EMMS) Resistance and Compliance (R&C) system was used to assess the pulmonary function of mice. After anesthesia was induced with intraperitoneally administration of xylazine (10 mg kg^−1^) and ketamine (100 mg kg^−1^), the mice underwent tracheostomy and were mechanically ventilated at a rate of 250 breaths per minute with a tidal volume of 250 mL. The mice were then moved to the EMMS R&C system to measure average airway resistance and dynamic lung compliance.

### Histological Examination

Flushed lung tissues were fixed in 10% neutral‐buffered formalin for 24 h prior to paraffin embedding. Five‐micrometers slices were cut and placed on a glass slide, followed Hematoxylin‐eosin (H & E) staining. Five random areas of the lung sections were selected to assess lung injury severity using the American Thoracic Society (ATS) lung injury score system.^[^ ^48^ ^]^

### Bronchoalveolar Lavage Fluid Analysis

After anesthetization, the lungs of the mice were lavaged with 1 mL PBS for BALF collection, which were centrifuged at 400 g for 10 min to isolate the cells. The number of leukocytes was calculated after removing erythrocytes. BALF supernatants were separated and stored at −80 °C for further examination. The total protein level in BALF was analyzed using the bicinchoninic acid (BCA) protein assay kit. The levels of cytokines in BALF were measured using ELISA kits.

### Evans Blue Dye Permeability Measurement

Thirty minutes after intravenously injecting 1.5 mL 0.5% Evans blue dye via jugular vein, lung tissues were continuously perfused with PBS to completely remove of the dye from pulmonary circulation. Subsequently, lungs were homogenized in formamide and centrifuged at 3000 rpm for 20 min, the supernatant was obtained to examine the Evans blue dye extravasation by spectrophotometry at 620 nm.

### Human Samples Collection

Peripheral blood samples of 97 ARDS patients and 72 healthy donors were collected. The peripheral blood of the healthy controls was provided by the Physical Examination Center of Xuzhou Medical University. The peripheral blood of ARDS patients were provided by the ICU of the Affiliated Hospital of Xuzhou Medical University and Shanghai Pulmonary Hospital. Lung tissues from three patients with pulmonary hypertension complicated by pneumonia and two patients with interstitial lung disease complicated by pneumonia after lung transplantation. Lung tissues distant from the tumor site was collected from five early‐stage lung cancer patients who had undergone surgical resection as healthy controls. Lung samples were minced and digested (RPMI 1640, 225U ml^−1^ collagenase Type I, 0.2 mg ml^−1^ DNase I) in a gentle MACS c‐tube. Samples were partially dissociated by a gentle MACS dissociator (Miltenyi, German). Cells were filtered through a 40‐µm strainer and treated with erythrocyte lysis buffer for 5 min on ice to remove red blood cells.

All specimens were obtained under the guidance of the U.S. Health Insurance Portability and Accountability Act (HIPAA) protocol and supervised by the ethics committee of the hospital (K23‐314Z).

### Mouse Single‐Cell Isolation

Sigle‐cell suspensions were obtained from mouse lung tissues as published.^[^ ^5^, ^49^ ^]^ Flushed lung tissues were minced into 1–2 mm pieces and digested with 5 ml digestive fluid (RPMI 1640, 1 mg ml^−1^ collagenase I, 30 µg ml^−1^ DNase) at 37 °C for 30 min. Mixtures were pipetted through a 40 µm disposable cell strainer and then centrifuged to isolate cells, which were then treated with erythrocyte lysis buffer on ice to remove red blood cells.

Spleen single‐cell isolation was as follows: 1) spleens were homogenized using the plunger of a 1 ml syringe; 2) The mixture was rinsed and filtered through a 40‐µm disposable strainer. 3) Cells were isolated after centrifugation and then subjected to red blood cell lysis on ice to remove red blood cells.

After washing with PBS, the cell suspensions were maintained on ice for subsequent analysis.

### Single‐Cell RNA Sequencing and Data Analysis

Lungs tissues were dissociated from three LPS‐treated WT mice and three LPS‐treated Cxcr1^i△DC/i△DC^ mice, as well as one PBS‐treated WT mouse and one PBS‐treated Cxcr1^i△DC/i△DC^ mouse. BD Rhapsody scRNA‐Seq platform was utilized to obtain the transcriptomic information of single cells as previously reported.^[^ ^50^, ^51^, ^52^ ^]^ Briefly, a single cell suspension was randomly distributed on BD Rhapsody cartridges. Beads were loaded onto cartridges to pair each single cell in a microwell. After washing the cartridges, cells were lysed and beads were collected for reverse transcription. Whole transcriptome libraries were established using the BD Rhapsody system (BD Biosciences).

Cells with an expressed gene count of <2% or >98% were excluded. Moreover, those containing >10% of reads corresponded to mitochondrial genes were filtered. The SingleR R package and well‐known cell‐specific marker genes were simultaneously used to annotate each cell cluster. Significant DEG was identified by two criteria: i, an average log_e_ fold‐change ≥0.25; ii, adjusted p value <0.05.

Pseudotime trajectories of differentiation were performed by Monocle2 R package. Pseudotime analysis was conducted to order cells along a calculated trajectory that could infer the transcriptional changes based on asynchronous manners in biological processes of each cell.

The CellPhoneDB R package was used to identify and visualize intercellular communications. The interaction intensity based on ligand‐receptor pairs between all cell types was determined using a permutation test and the p value across the ligand‐receptor was calculated using the Bonferroni multiple‐ pair comparison test. Only ligand‐receptor pairs expressed in >10% of the cells were regarded as potential molecular partner that mediated cell‐cell interactions.

### Flow Cytometry Analysis

Cells were diluted to 1×10^6^/100 µL stained with the following fluorochrome‐conjugated antibodies (Abs): CD45‐PerCP/Cyanine5.5 (Dilution 1:200, #157 208, Biolegend), CD11c‐PE (Dilution 1:200, #117 308, Biolegend), MHCII‐APC (Dilution 1:200, #107 614, Biolegend), MHCII‐FITC (Dilution 1:200, #107 606, Biolegend), CD11b‐PE/Cyanine7 (Dilution 1:200, #101 216, Biolegend), CD103‐ Brilliant Violet 510 (Dilution 1:200, #121 423, Biolegend), CD8a‐Brilliant Violet 510 (Dilution 1:200, #100 752, Biolegend), Ly6C‐FITC (Dilution 1:200, #128 006, Biolegend), CD4‐FITC (Dilution 1:200, #100 406, Biolegend), CD25‐PE (Dilution 1:200, #102 008, Biolegend), Foxp3‐Alexa Fluor647 (Dilution 1:200, #126 408, Biolegend), IL‐17A‐APC (Dilution 1:200, #506 916, Biolegend), CXCR1‐APC (Dilution 1:200, #FAB8628A‐100, R&D), CD45‐FITC (Dilution 1:200, #304 006, Biolegend), CD11c‐PE (Dilution 1:200, #337 205, Biolegend), HLA‐DR‐PE/Cyanine7 (Dilution 1:200, #327  018, Biolegend), CD141‐Brilliant Violet 421™ (Dilution 1:200, #344 114, Biolegend), CD1c‐PerCP/Cyanine5.5 (Dilution 1:200, #331 514, Biolegend), CD14‐APC (Dilution 1:200, #301 808, Biolegend), CXCR1‐APC (Dilution 1:200, #320 612, Biolegend), and live/dead‐zombie NIR (Dilution 1:1000, #423 105, Biolegend).

### DC‐T Cell Coculture

DCs were isolated from the lungs and spleens of PBS‐ or LPS‐ treated WT and Cxcr1^−/−^ mice using a BD FACS ARIA‐II cell sorter (BD Biosciences). Naïve T cells were purified from spleens of WT mice via magnetic bead cell sorting (#480 040, Biolegend). DCs were co‐cultured with Naïve T cells at a ratio of 1:10 (naïve T: 2×10^5^ cells/well, DCs: 2×10^4^ cells/well) with plate‐bound anti‐CD3 (2 µg ml^−1^) and anti‐CD28 (2 µg ml^−1^) in a 96‐well U‐bottom plate. For Treg differentiation, neither factors nor neutralizing Abs were added in the RPMI medium. For Th17 differentiation, RPMI medium was additionally supplemented with anti‐IFNγ (10 µg ml^−1^), and anti‐Il‐4 (10 µg ml^−1^). In optimized Treg differentiation conditions, IL‐2 (10 ng ml^−1^) and TGF‐β (2.5 ng ml^−1^) were additionally supplemented in the medium. After 3 days of incubation, IL‐10 and IL‐17A in the supernatant were examined using ELISA kits. Other inflammatory cytokines were measured by 36‐Plex ProcartaPlex 1A. Luminex assay (EPX360‐26092‐901, Thermo). The percentage of Tregs (CD4^+^ CD25^+^ Foxp3^+^) and Th17 (CD4^+^ IL‐17A^+^) cells was analyzed via flow cytometry.

### Adoptive Transfer

Three subpopulations of DCs (Ly6C^+^ cDC2s, Ly6C^−^ cDC2s, cDC1s) were isolated from the spleens of LPS‐treated Cxcr1^−/−^ or WT mice via FACS sorting (BD Biosciences). A total of 200000 cells of each subpopulation were injected i.v. into WT C57BL/6J mice with equal volume of PBS as control. After 24 h, the recipient mice were challenged with LPS as described above. Three days after LPS treatment, these mice were humanely sacrificed for subsequent examination.

### RNA Isolation and Quantitative Real‐Time PCR (q‐PCR)

Total RNA was isolated using TRIzol reagent (Invitrogen) and cDNA was synthesized using ABScript lll RT MasterMix (Abclonal). Q‐PCR was performed on cDNA with UltraSYBR One Step RT‐qPCR Kit (CWBIO). Relative fold expression of individual genes was calculated using the double delta Ct method, with β‐actin as a housekeeping gene for endogenous control. Primer information was presented in Table S1 (Supporting Information).

### Western Blot Analysis

Proteins were separated by sodium dodecyl sulfate‐polyacrylamide gel electrophoresis (SDS‐PAGE) and transferred to 0.45 mm polyvinylidene fluoride (PVDF) membranes (MilliporeSigma). After blocking with 5% non‐fat dry milk for 30 min, the membranes were separately incubated, overnight at 4 °C, with the appropriate primary antibodies. Subsequently, the membranes were incubated with the corresponding secondary antibody for 120 min at room temperature. Finally, the immunoreacted protein bands were visualized.

### Quantification and Statistical Analysis

Statistical analyses were performed using R (v4.2.2) or GraphPad Prism (v.9) software. Flow cytometry data were quantified using the FlowJo (v10) software. Details were provided in the figure legends. A p‐value of <0.05 was considered statistically significant.

## Conflict of Interest

The authors declare no conflict of interest.

## Author Contributions

S.L., W.Z., Y.W., and H.Q. contributed equally to this work. S.L., X.L., and C.C. performed conceptualization, data curation, formal analysis investigation, methodology, software, visualization, wrote the original draft, and wrote, ‐reviewed, and edited the draft; W.Z., Y.W., and H.Q. data curation, formal analysis investigation, methodology, and visualization. X.Q. performed data curation, formal analysis investigation, and visualization. X.H. performed conceptualization, data curation, formal analysis investigation, methodology, software, and visualization.

## Supporting information



Supporting Information

## Data Availability

The data that support the findings of this study are available from the corresponding author upon reasonable request.
