# Supplementary material for: CXCR1 Depletion in Ly6C+ cDC2 Alleviates Acute Lung Injury via Modulation of Th17/Treg Balance
Source: Adv Sci (Weinh). 2025 Aug 11;12(41):e06287. doi: 10.1002/advs.202506287 (PMC12591108; doi:10.1002/advs.202506287)

**Supplementary Table 1: Primer sequences used in this study**

| **Gene** | **Forward (5’-3’)** | **Reverse (5’-3’)** |
| --- | --- | --- |
| CXCR1 | TGCCTCAACCCCATCATCTAC | TAACACGATGACGTGCCAAGAA |
| β-ACTIN | CTCCATCCTGGCCTCGCTGT | GCTGTCACCTTCACCGTTCC |

**Supplementary Table 2: Characteristics of the healthy donors and ICU patients with ARDS.**

| **Variables** | **Healthy donors**  **(n=72)** | **Patients with ARDS (n=97)** |
| --- | --- | --- |
| **Gender** |  |  |
| Female | 41 (56.94%) | 53(54.64%) |
| Male | 31(43.06%) | 44(45.36%) |
| **Age, mean [95% CI] years** | 33 (25-41) | 52 (41-60) |
| **Type of ICU admission** |  |  |
| Medical |  | 61 (62.89%) |
| Surgical |  | 36 (37.11%) |
| **Mechanical ventilation** |  | 41 (42.27%) |
| **Major diseases** |  |  |
| Infection |  | 33(34.02%) |
| Respiratory failure |  | 12 (12.37%) |
| Cerebral apoplexy |  | 7 (7.22%) |
| Renal failure |  | 6 (6.19%) |
| Hepatic failure |  | 16 (16.49%) |
| Cancers |  | 12 (12.37%) |
| Others |  | 11 (11.34%) |

Abbreviations: ICU, intensive care unit

**
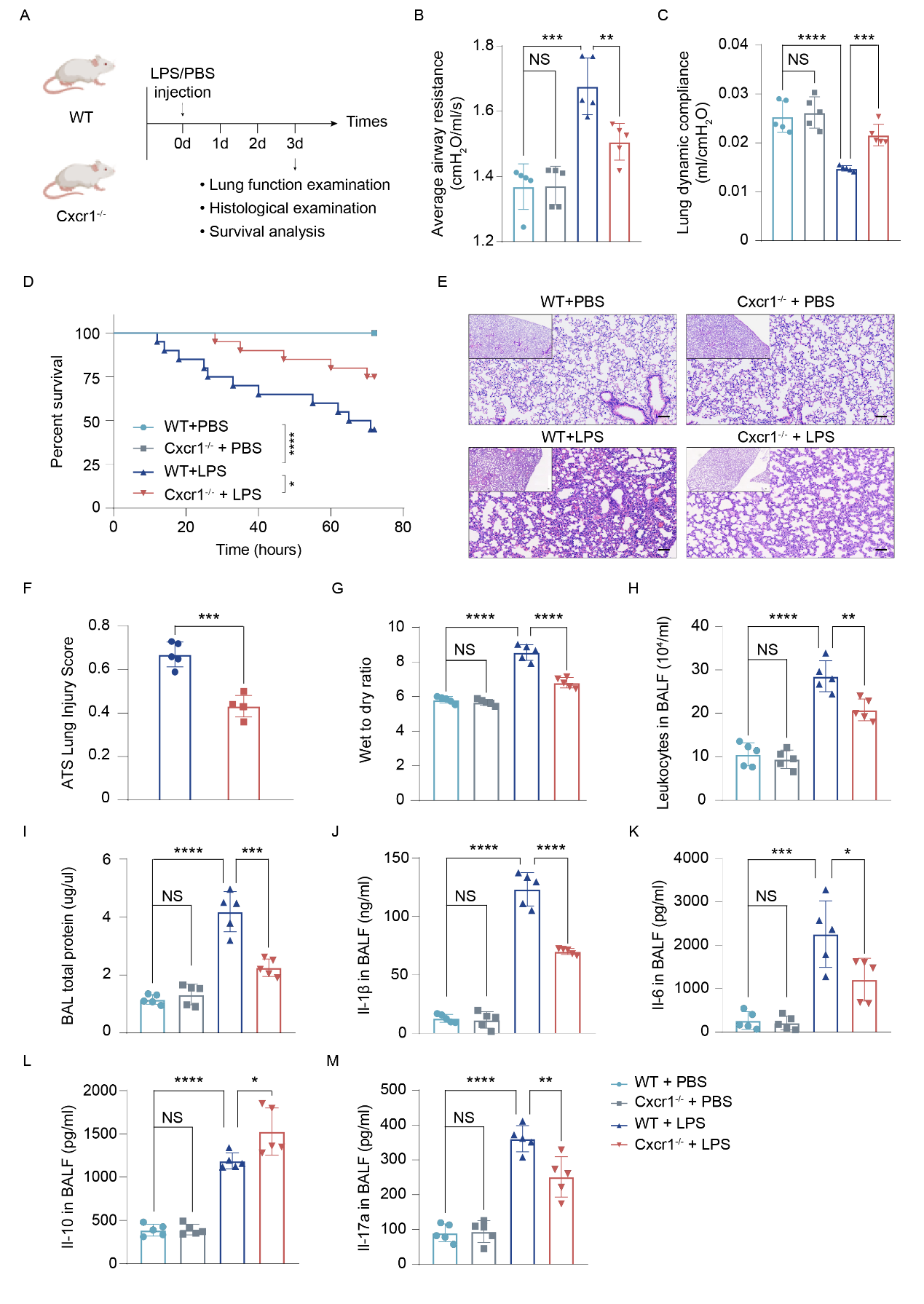
**

**Figure S1. Cxcr1 knockout mitigates LPS-induced ALI**

(A) Schematic diagram Cxcr1 knockout and LPS-induced ALI.

(B and C) Determination of the lung average airway resistance (B) and dynamic compliance (C) (n=5)

(D) Cxcr1 knockout leads to improved survival outcomes in LPS-treated mice (n = 20), log-rank test.

(E) Representative H&E staining of lung sections. Scale bars, 100 μm.

(F-I) Cxcr1 knockout reduces the lung injury score (F), lung wet/dry weight ratio (G), and the number of leukocytes (H) and total protein in BALF (I) (n = 5).

(J-M) The effect of Cxcr1 knockout on the levels of Il-1β (J), Il-6 (K), Il-10 (L), and Il-17a (M) in BALF (n = 5).

*p < 0.05, **p < 0.01, ***p < 0.001,****p < 0.0001, NS, p＞0.05 vs. the indicated group, one-way ANOVA followed by Tukey’s post hoc test.

**
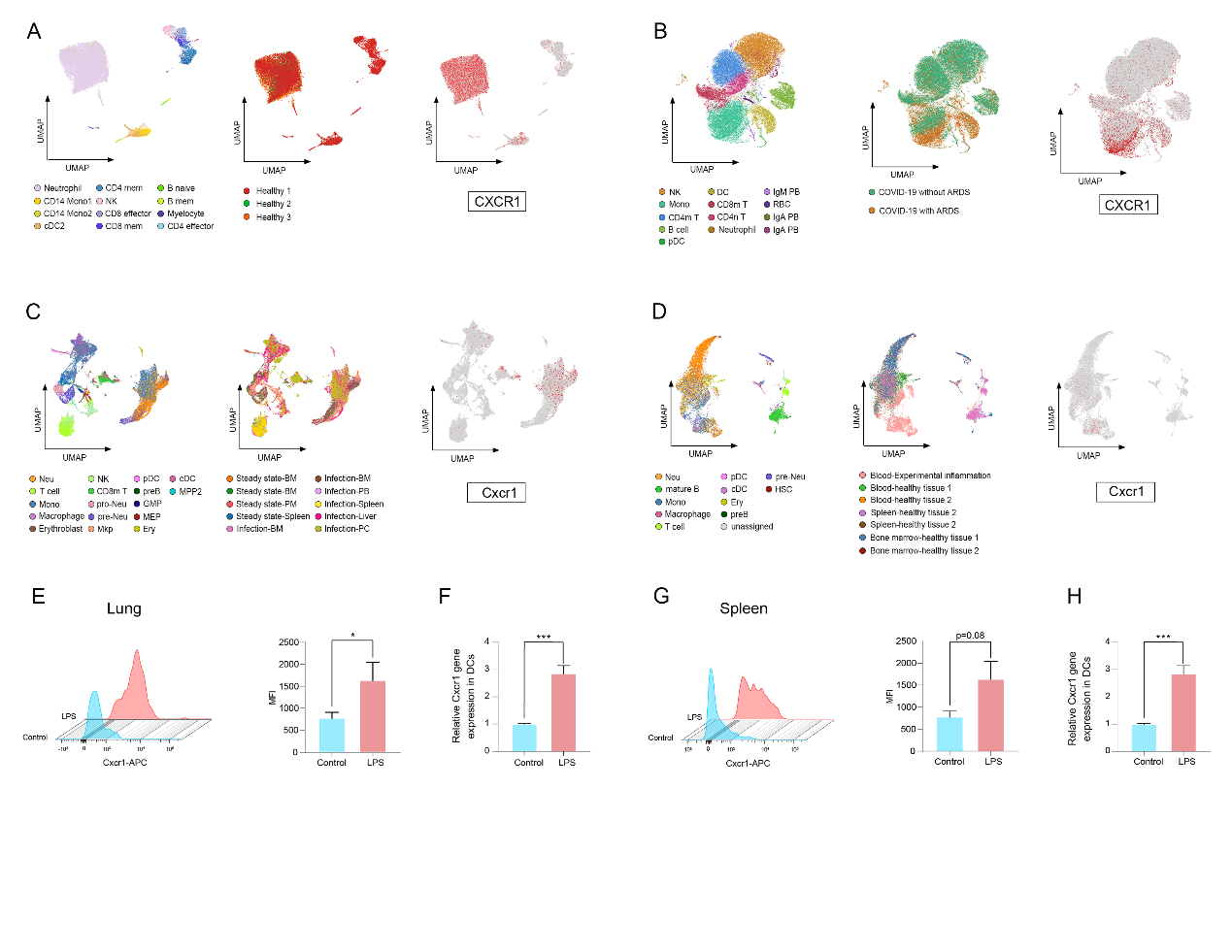
Figure S2. Cxcr1 is highly expressed in DCs and involved in various inflammatory disease including ARDS.**

(A) UMAP visualization of cell populations from peripheral blood samples of three healthy donors, and the expression of CXCR1 genes across cell populations (GSE137540).

(B) UMAP visualization of cells from peripheral blood mononuclear cells (PBMCs) of patients hospitalized for COVID-19 and healthy controls, and the expression of CXCR1 genes (GSE150728).

(C) UMAP visualization of cell populations from the bone marrow, peripheral blood, liver, and spleen tissues of the control and infected mice, and the expression of Cxcr1 genes (GSE137540).

(D) UMAP visualization of cell populations from the bone marrow, peripheral blood, and spleen tissues of healthy and inflammatory mice models, and the expression of Cxcr1 genes (GSE165276).

(E and F) Flow cytometry (E) and q-PCR determined Cxcr1 expression in DCs from lung tissues of PBS-treated and LPS-treated mice (n=5).

(G and H) Flow cytometry (G) and q-PCR (H) determined Cxcr1 expression in DCs from spleen tissues of PBS-treated and LPS-treated mice (n=5).

*p < 0.05, **p < 0.01, ***p < 0.001,****p < 0.0001, NS, p＞0.05, vs. the indicated group, Student’s T-test.


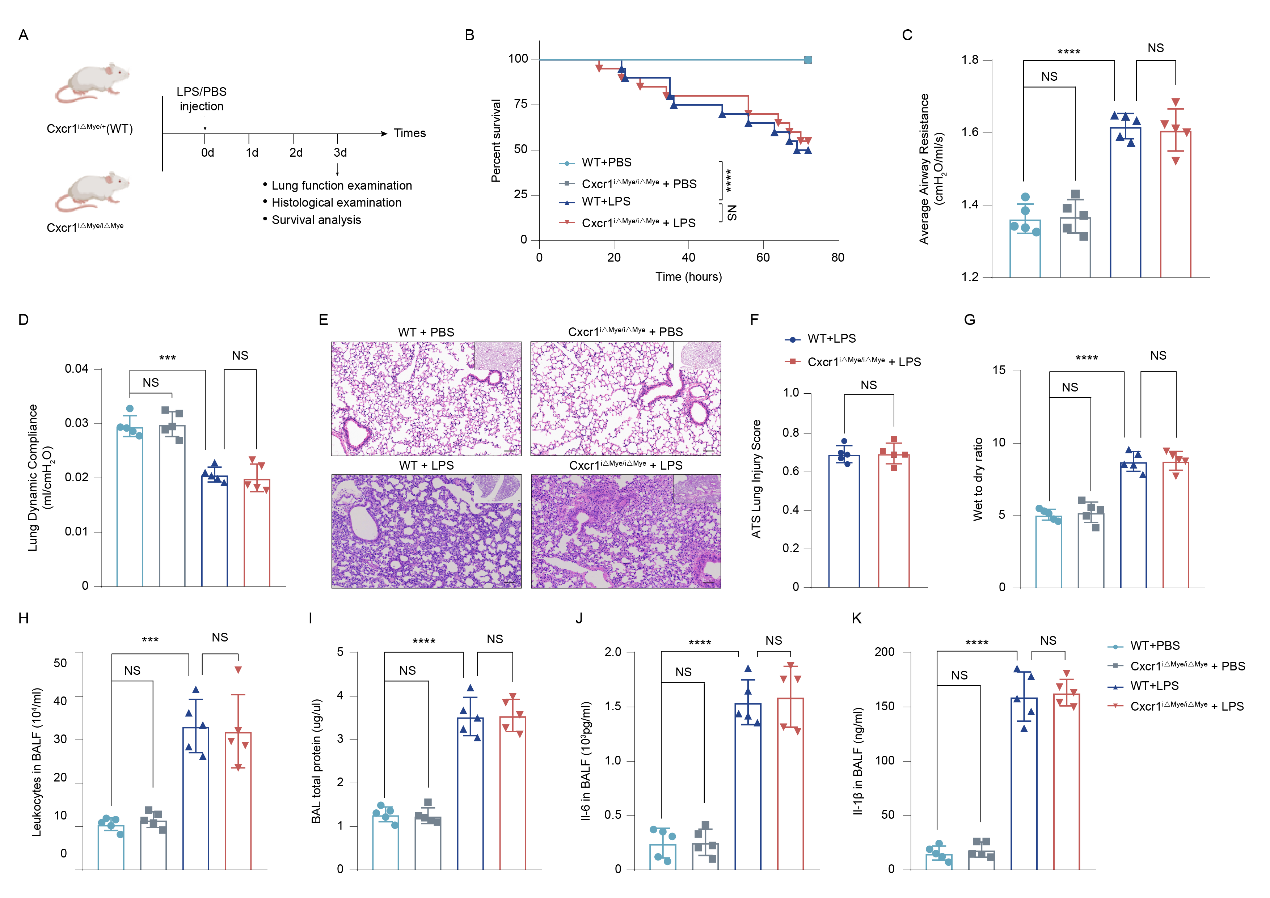


**Figure S3. Cxcr1 deficiency in myeloid cells has no impact on the progression of ALI.**

(A) Schematic diagram of inducible myeloid-specific depletion of Cxcr1 and LPS-induced ALI in mice.

(B) Cxcr1 deficiency in myeloid cells did not affect the survival outcomes of LPS-treated mice (n = 20), log-rank test.

(C and D) Determination of the lung average airway resistance (C) and dynamic compliance (D) (n = 5).

(E) Representative H&E staining of lung sections. Scale bars, 100 μm.

(F–I) The effect of Cxcr1 deficiency in myeloid cells on the lung injury score (F), lung wet/dry weight ratio (G), and number of leukocytes (H) and total protein (I) in BALF (n = 5).

(J and K) The effect of Cxcr1 deficiency in myeloid cells on the Il-6 (J) and Il-1β (K) in BALF (n = 5).

*p < 0.05, **p < 0.01, ***p < 0.001,****p < 0.0001, NS, p＞0.05 vs. the indicated group, one-way ANOVA followed by Tukey’s post hoc test.

**
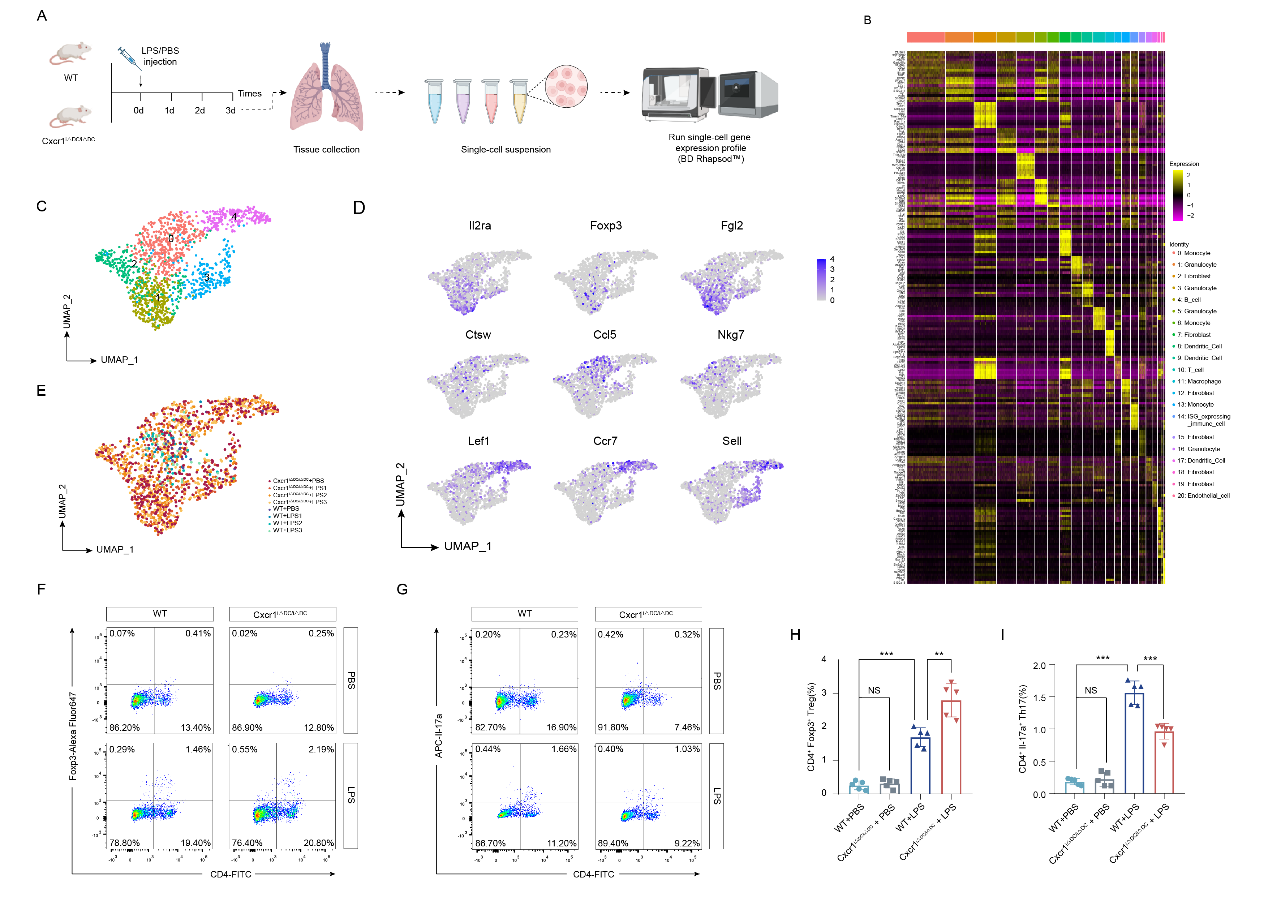
**

**Figure S4. Cxcr1 deficiency in DCs regulates Th17/Treg balance in vivo.**

(A) Schematic of sample collection for scRNA-seq analysis.

(B) Heatmap showed the top 10 DEGs of each cluster.

(C) UMAP visualization of T cell subsets.

(D) Feature plots displaying the expressions of known T cells marker genes.

(E) UMAP visualization of T cell populations colored according to sample source.

(F-I) Flow cytometry showing the proportion of splenic CD4^+^Foxp3^+^ Tregs (F and H) and CD4^+^IL-17a^+^ Th17 cells (G and I) in PBS- or LPS-treated WT and Cxcr1^i△DC/i△DC^ mice (n=5).

*p < 0.05, **p < 0.01, ***p < 0.001,****p < 0.0001, NS, p＞0.05 vs. the indicated group, one-way ANOVA followed by Tukey’s post hoc test.

**
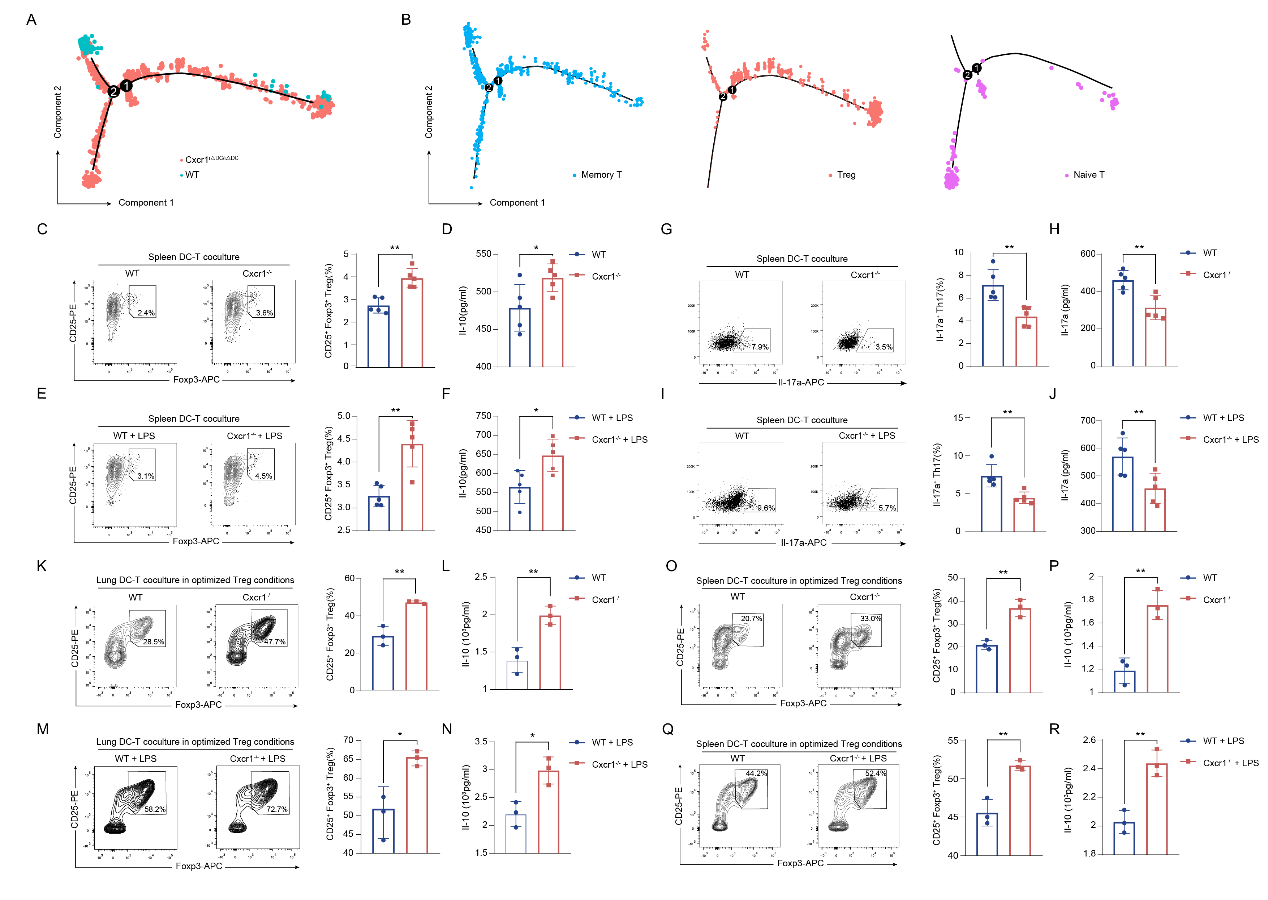
Figure S5. Cxcr1 deficiency in DCs promotes Treg but constrain Th17 differentiation.**

(A and B) The developmental trajectory of T cells inferred by Monocle2, colored by sample sources and subsets.

(C and E) Flow cytometry analysis showing the proportion of CD25^+^Foxp3^+^ Tregs from spleen DC-naïve T cells co-culture system (n=5).

(D and F) ELISA analysis detects Il-10 levels in the co-culture medium (n=5).

(G and I) Flow cytometry analysis showing the proportion of Il-17a^+^ Th17 from spleen DC-naïve T cells co-culture system (n=5).

(H and J) ELISA analysis detects Il-17a levels in the co-culture medium (n=5).

(K and M) Flow cytometry showing the proportion of CD25^+^Foxp3^+^ Tregs from lung DC-naïve T cells co-culture system in optimized Treg differentiation conditions (n=3).

(L and N) ELISA analysis detects Il-10 levels in the co-culture medium (n=3).

(O and Q) Flow cytometry showing the proportion of CD25^+^Foxp3^+^ Tregs from spleen DC-naïve T cells co-culture system in optimized Treg differentiation conditions (n=3).

(P and R) ELISA analysis detects Il-10 levels in the co-culture medium (n=3).

*p < 0.05, **p < 0.01, ***p < 0.001,****p < 0.0001, NS, p＞0.05 vs. the indicated group, Student’s T-test.

**
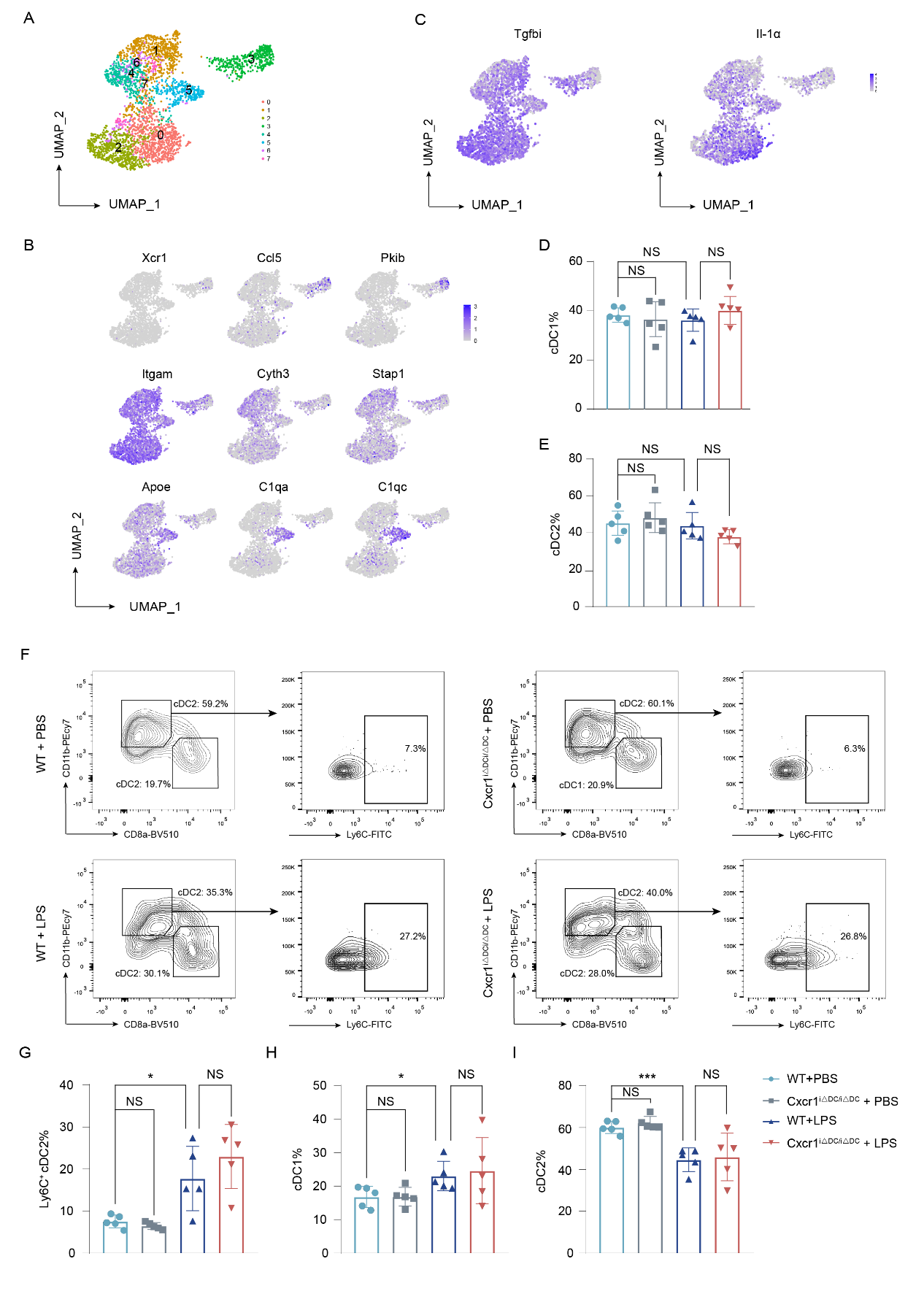
**

**Figure S6. Ly6C^+^ cDC2s aggregated in inflammatory settings without affection of Cxcr1**

(A) UMAP visualization of DC subsets.

(B) Feature plots displaying the expressions of known DC marker genes.

(C) Feature plots displaying the expressions of Tgfbi and Il-1α genes across DCs.

(D and E) The percentage of cDC1s (D) and cDC2s (E) in the lungs of PBS- or LPS-treated WT and Cxcr1^i△DC/i△DC^ mice (n=5).

(F) Flow cytometry analysis showing the proportion of splenic Ly6C^+^ cDC2s, cDC1s, and cDC2s in PBS- or LPS-treated WT and Cxcr1^i△DC/i△DC^ mice (n=5).

(G-I) Pooled data of splenic Ly6C^+^ cDC2 (G), cDC1 (H), and cDC2 (I) percentage presented from (F) (n=5).

*p < 0.05, **p < 0.01, ***p < 0.001,****p < 0.0001, NS, p＞0.05 vs. the indicated group, one-way ANOVA followed by Tukey’s post hoc test.

**
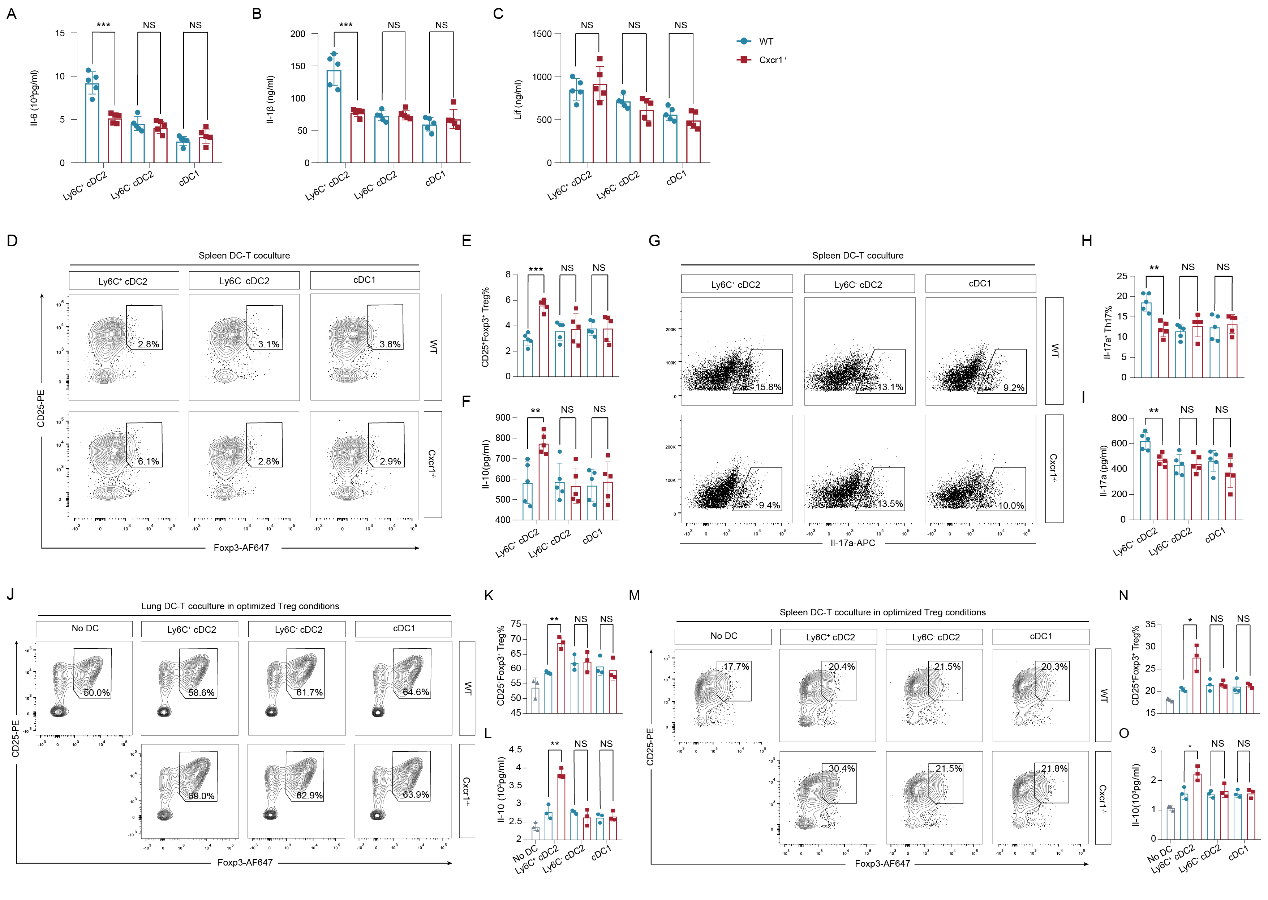
**

**Figure S7.** **Cxcr1 deficiency specifically converts the pro-inflammatory phenotype of Ly6C^+^ cDC2 in vitro.**

(A-C) Levels of Il-6 (A), Il-1β (B), and Lif (C) in supernatants from individually cultured Ly6C^+^ cDC2s, Ly6C^-^ cDC2s, and cDC1s isolated from the spleens of WT and Cxcr1^-/-^ mice treated with LPS (n=5).

(D and E) The impact of Cxcr1 deficiency on the activity of splenic Ly6C^+^ cDC2s, Ly6C^-^ cDC2s, and cDC1s in regulating the differentiation of naïve T cells into Tregs (n=5).

(F) Il-10 levels in the co-culture medium (n=5).

(G and H) The impact of Cxcr1 deficiency on the activity of splenic Ly6C^+^ cDC2s, Ly6C^-^ cDC2s, and cDC1s in inducing the differentiation of naïve T cells into Th17 cells (n=5).

(I) Il-17a levels in the co-culture medium (n=5).

(J and K) Flow cytometry showing the proportion of CD25^+^Foxp3^+^Tregs from lung DC-naïve T cells co-culture system in optimized Treg differentiation conditions (n=3).

(L) Il-10 levels in the co-culture medium (n=3).

(M and N) Flow cytometry showing the proportion of CD25^+^Foxp3^+^Tregs from spleen DC-naïve T cells co-culture system in optimized Treg differentiation conditions (n=3).

(O) Il-10 levels in the co-culture medium (n=3).

*p < 0.05, **p < 0.01, ***p < 0.001,****p < 0.0001, NS, p＞0.05 vs. the indicated group, Student’s T-test.


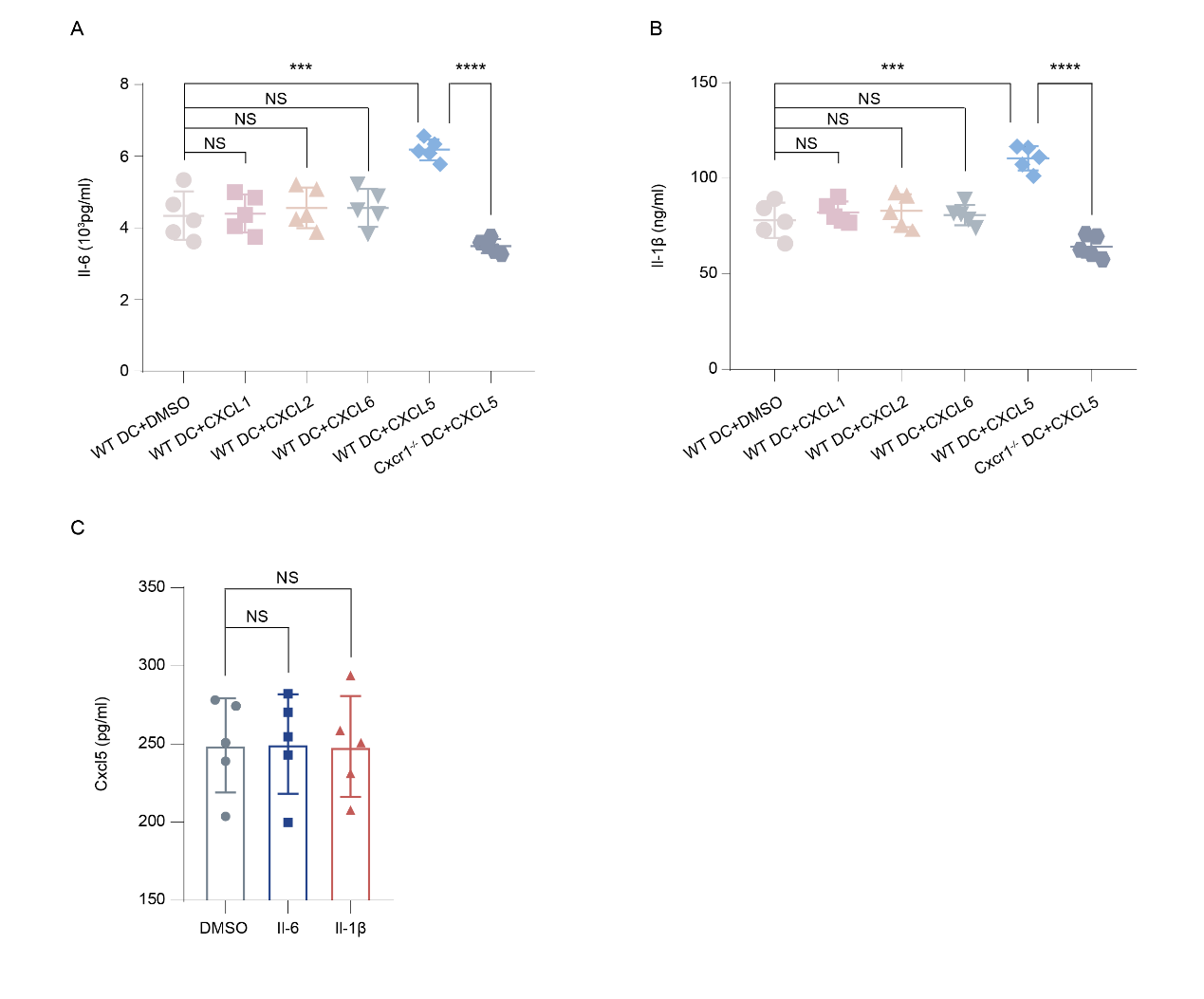


**Figure S8.** **Cxcl5 serves as the primary ligand activating Cxcr1 signalling in mouse DCs.**

(A and B) ELISA analysis was used to evaluate the impact of Cxcr1 ligands on the release of Il-6 (A) and Il-1β (B) of DCs from the lungs (n=5).

(C) ELISA analysis was used to evaluate the impact of Il-6 and Il-1β on Cxcl5 production of T cells from lungs (n=5).

*p < 0.05, **p < 0.01, ***p < 0.001,****p < 0.0001, NS, p＞0.05 vs. the indicated group, one-way ANOVA followed by Tukey’s post hoc test.

Source Figure 1


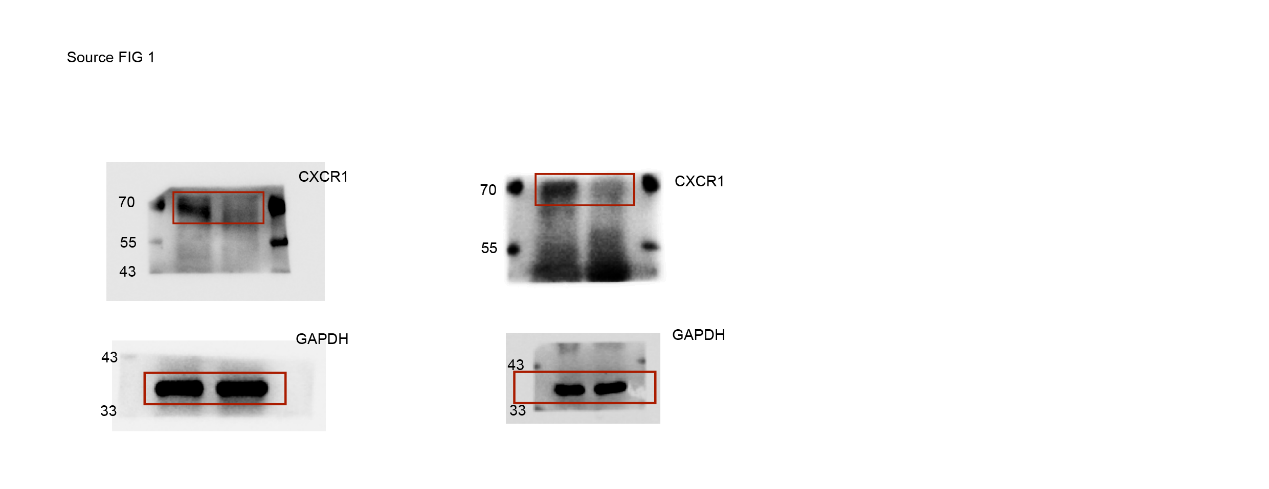


Source Figure 7A


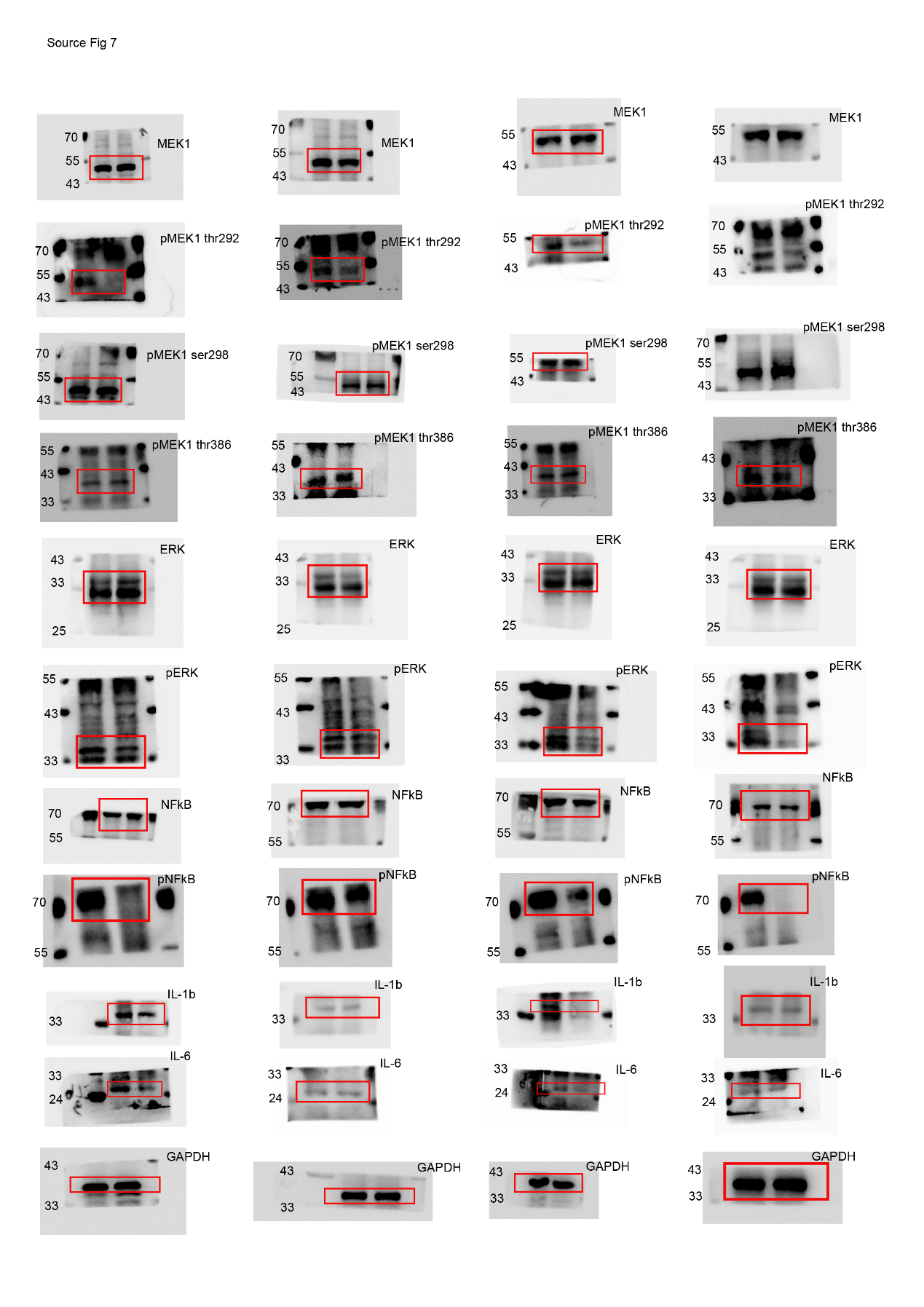


Source Figure 7E


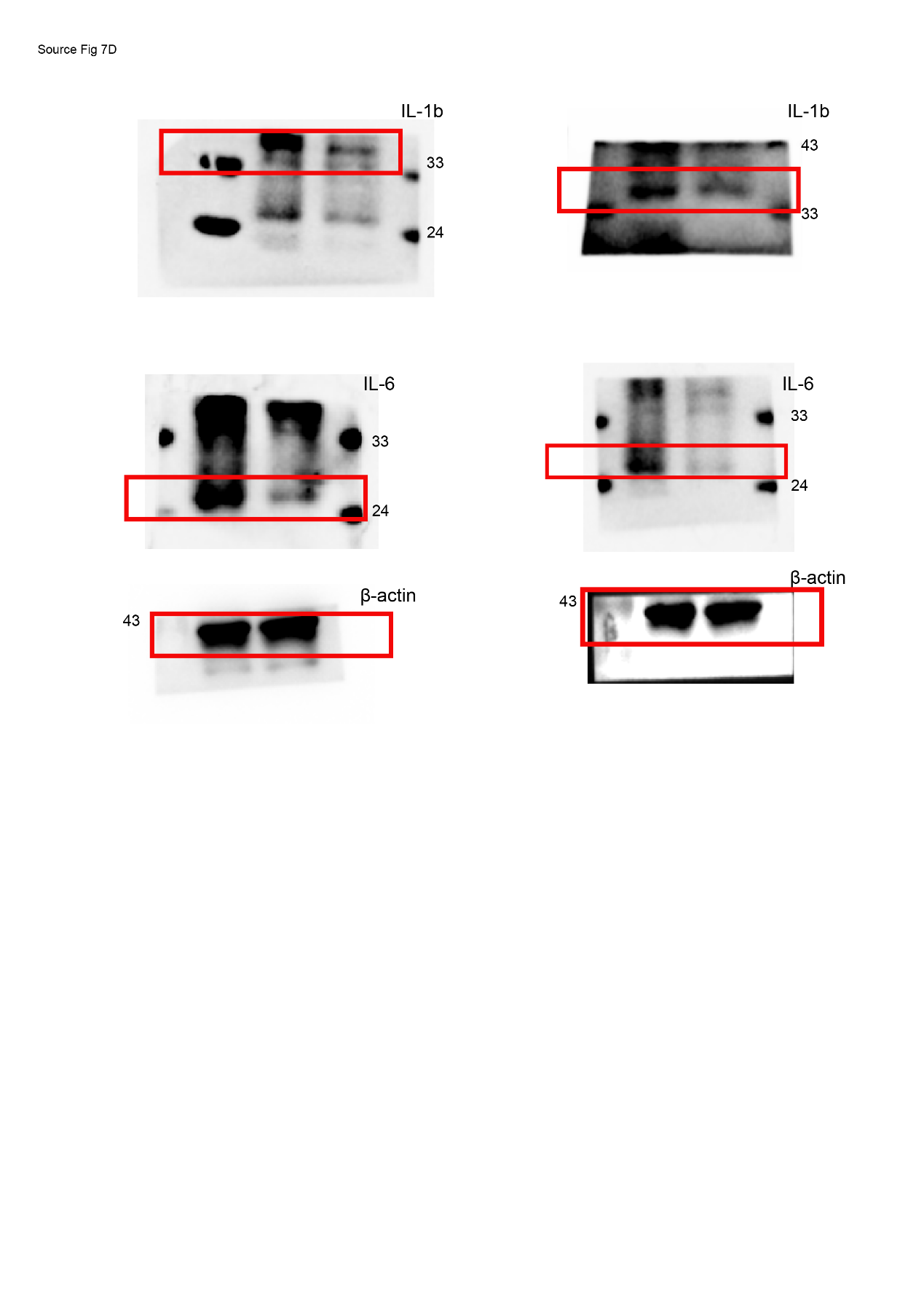

Supplement: Supplementary file 1 — Supporting Information [file ADVS-12-e06287-s001.docx]
